# Profile of the Bioactive Compounds, Minerals, Antioxidant and Antimicrobial Properties of Coloured Cherry Tomatoes at Different Stages of Ripeness

**DOI:** 10.3390/foods15173083

**Published:** 2026-08-31

**Authors:** Elena Coyago-Cruz, Gabriela Méndez, Josselyn Tenelema, Micaela Benalcázar, Jorge Heredia-Moya

**Affiliations:** 1 Carrera de Ingeniería en Biotecnología, Universidad Politécnica Salesiana, Sede Quito, Campus El Girón, Quito 170143, Ecuador; 2 Centro de Investigación Biomédica (CENBIO), Facultad de Ciencias de la Salud Eugenio Espejo, Universidad UTE, Quito 170527, Ecuador

**Keywords:** functional foods, nutraceuticals, *Solanum lycopersicum*, MIC

## Abstract

Cherry tomatoes are recognised for their nutritional value and for being an important source of bioactive compounds. The aim was to evaluate the effect of ripening on the commercial quality, mineral composition, bioactive compounds, and antioxidant and antimicrobial activity of five differently coloured cherry tomato accessions. The bioactive compounds were quantified using rapid-resolution liquid chromatography, and antioxidant activity was measured by microplate spectrophotometry. The results showed significant differences among accessions and ripeness stages. Thus, the highest concentrations were 5263.7 mg/100 g DW for potassium, 469.3 mg/100 g DW for vitamin C, and 5866.5 mg/100 g DW for total organic acids, 46.2 mg/100 g DW for anthocyanins, 1073.7 mg/100 g DW for total carotenoids, 1447.7 mg/100 g DW for total phenolic compound, and 4.7 mmol TE/100 g DW for DPPH antioxidant activity. The crude ethanolic extracts showed moderate inhibitory effects against *Escherichia coli*, *Staphylococcus aureus*, *Pseudomonas aeruginosa* and *Streptococcus mutans*, with minimum inhibitory concentrations ranging from 1.95 to 83.96 mg/mL. However, no activity was observed against *Candida albicans* or *Candida tropicalis*. Taken together, these results demonstrate the high nutritional and functional potential of cherry tomatoes grown in Ecuador.

## 1. Introduction

The tomato (*Solanum lycopersicum* L.) is native to South America, where it was domesticated primarily in the Andean regions of Ecuador and Peru. Following the Spanish conquest, it was introduced to the Iberian Peninsula, from where it spread to the rest of the world, giving rise to an extensive process of genetic improvement that has produced a wide variety of cultivars, sizes and colours [1]. Within this species, the cherry tomato (*Solanum lycopersicum* var. cerasiforme) is recognised as the ancestor of large-fruited varieties and is currently valued for its colour diversity and nutritional value [2].

Wild species, large-fruited tomatoes and cherry tomatoes together constitute one of the most important horticultural crops for human consumption and the agri-food industry. Currently, nutritional trends have shifted from the concept of ‘eating enough’ towards that of ‘eating healthily’, increasing interest in varieties with superior nutritional characteristics. In addition to organoleptic quality, the tomato’s versatility for fresh consumption and industrial use has contributed to its widespread acceptance [3].

Beyond their contribution of essential nutrients, the importance of the tomato in the human diet lies in its richness in bioactive compounds. These include vitamin C and vitamin E, as well as various organic acids, including citric, malic, oxalic and tartaric acids, which contribute to the fruit’s characteristic flavour and freshness [2]. Their functional value is also associated with a complex carotenoid profile, dominated by lycopene and β-carotene, as well as a wide variety of phenolic compounds and flavonoids, notably rutin and quercetin [4].

In recent years, the growing demand for foods with functional properties has increased interest in cherry tomatoes of different colours. Varieties with red, yellow, green, black, or purple pigmentation exhibit distinct phytochemical profiles due to differences in the accumulation of carotenoids, chlorophylls, anthocyanins, and phenolic compounds. Furthermore, the development of varieties with high anthocyanin content has enabled the production of dark-coloured fruits with potential nutraceutical properties [5]. In these materials obtained through conventional breeding programmes, anthocyanins accumulate mainly in the epicarp rather than in the pulp. Furthermore, the synthesis of these pigments depends largely on light intensity and temperature, which is why the shaded areas of the fruit tend to exhibit less purple pigmentation [6].

The nutritional and functional quality of the tomato is strongly influenced by the variety and the fruit’s ripeness. During ripening, profound physiological and biochemical changes occur that affect size, texture, colour, flavour, and nutritional composition. These processes alter the concentrations of sugars, organic acids, carotenoids, phenolic compounds, vitamins, and minerals. However, the magnitude and direction of these changes depend largely on each cultivar’s genetic background, resulting in substantial differences among varieties [7,8].

These secondary components are primarily responsible for the fruit’s antioxidant activity and act as scavengers of reactive oxygen species (ROS) [4]. This capacity, commonly measured using assays such as DPPH, ABTS, FRAP, and ORAC, correlates directly with the concentration of carotenoids and other compounds that prevent neurodegenerative and cardiovascular diseases, as well as certain types of cancer, by protecting cells from oxidative damage. Consequently, tomato consumption has been associated with various health benefits, including reduced susceptibility to cardiovascular, neurodegenerative and intestinal diseases, as well as an improved immune response and greater skin protection [1].

Furthermore, the study of antimicrobial activity is a critical priority in human health due to the growing crisis of resistance to conventional drugs and the substantial impact of infections on global mortality. Thus, fungal infections cause approximately 1.7 million deaths per year, a rate comparable to that of tuberculosis. Species such as *Candida* are the fourth leading cause of bloodstream infections worldwide, with a mortality rate of up to 40%. Furthermore, certain populations, such as the elderly, hospitalised patients and immunocompromised individuals, are particularly vulnerable to these infections, which can range from superficial conditions to life-threatening diseases [9]. In this context, some existing drugs have hepatotoxic and nephrotoxic effects; searching for safer and more effective natural, plant-derived alternatives is a viable option. Thus, various studies have shown that the phenolic compounds present in the fruit can inhibit the growth of bacteria and fungi [4,10].

Although numerous studies have assessed the influence of ripening on the chemical composition of tomatoes, most have focused on traditional commercial cultivars or specific groups of bioactive compounds. This situation is particularly evident in accessions grown under open-field conditions in Ecuador, where the diversity of local germplasm has received comparatively less scientific attention and most studies have focused on varieties grown in Europe, Asia and Africa. Therefore, this study aimed to compare the commercial quality, mineral composition, vitamin C, organic acids, carotenoid phenolic compound profiles, antioxidant capacity (ABTS and DPPH), and antibacterial activity (*Escherichia coli* ATCC 8739, *Staphylococcus aureus* ATCC 6538P, *Pseudomonas aeruginosa* ATCC 9027 and *Streptococcus mutans* ATCC 25175) and antifungal assays (*Candida albicans* ATCC 1031 and *Candida tropicalis* ATCC 13803) of five differently coloured cherry tomato accessions at three ripeness stages under open-field conditions in the Andean region of Ecuador. The contribution of this study lies in the integrated comparison of five non-authenticated cherry tomato accessions across three ripening stages, which allowed the identification of accession-dependent and ripening-dependent patterns in physicochemical characteristics, mineral composition, bioactive compounds, in vitro antioxidant capacity, and antimicrobial responses.

## 2. Materials and Methods

### 2.1. Determination of Market Quality

**Figure 1:**
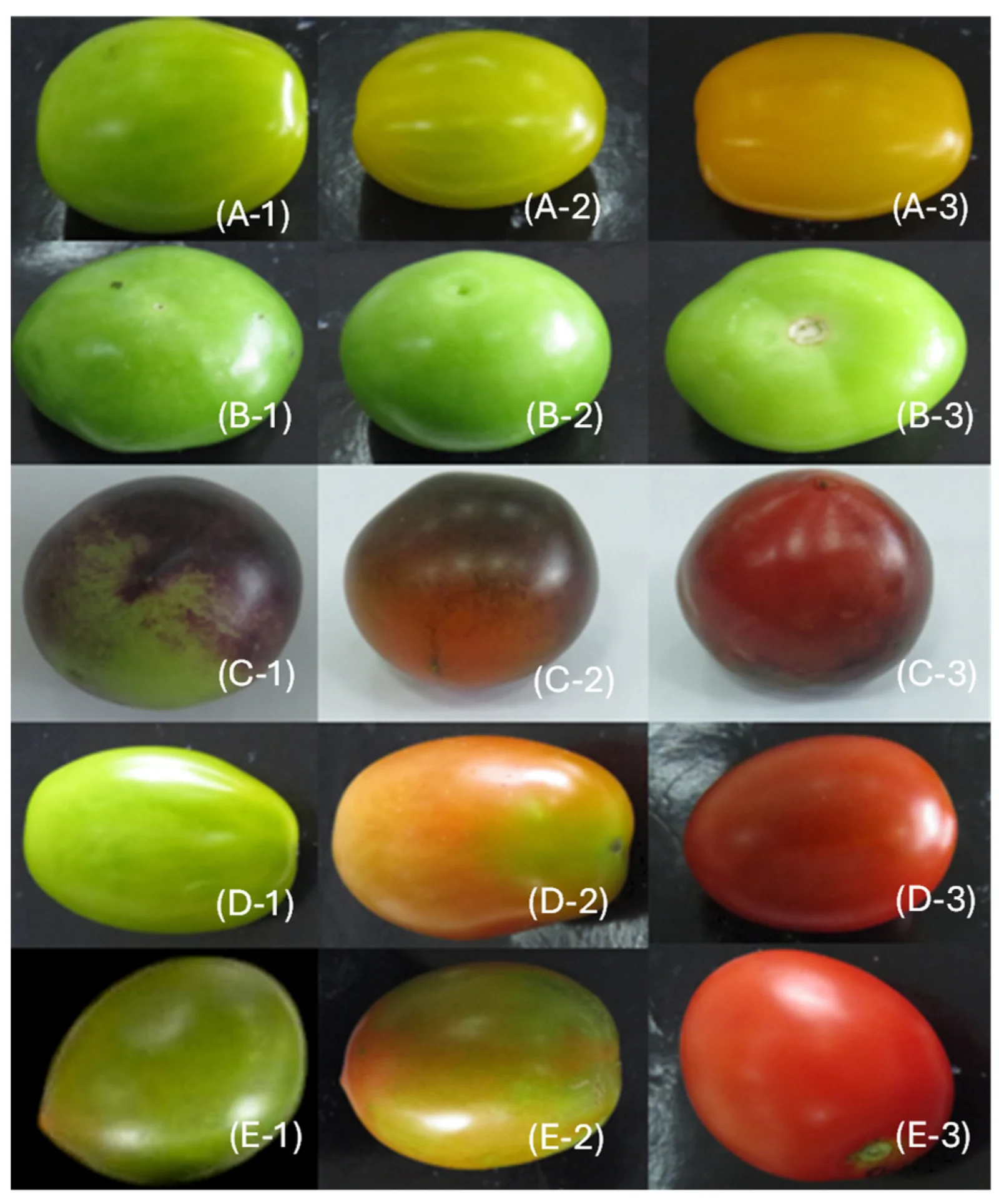
Cherry tomato (*Solanum lycopersicum* L.) accessions evaluated at three ripeness stages: (**A**) ‘Yellow oval’; (**B**) ‘Round green’; (**C**) ‘Round black’; (**D**) ‘Red oval’; (**E**) ‘Heart’. Ripeness stages: (**1**) agronomic maturity; (**2**) 50% of the final fruit colour; (**3**) 100% of the final fruit colour. Accession denominations were assigned according to their characteristic fruit colour and shape.

For this study, five cherry tomato accessions were selected, distinguished by the shape and colour pattern of their fruits (Figure 1). The ‘Yellow oval’ accession produced oval-shaped fruits that changed colour from green to yellow during ripening. The ‘Round green’ accession produced round, green fruits that retained this colour throughout ripening, with slight variations in green intensity. The ‘Round black’ accession was characterised by round fruits with a combination of purple and green hues in the early stages, which evolved into a mixture of purple and red as ripening progressed. The ‘Red oval’ accession produced oval-shaped fruits whose colour gradually changed from green to red during the ripening process. Finally, the ‘Heart’ accession produced heart-shaped fruits that changed from green when unripe to red at physiological maturity. The names of these accessions are commonly used to market these cherry tomatoes in Pichincha, Ecuador.

The accessions were grown in open fields in the province of Pichincha, in Ecuador’s Andean region. This region enjoys approximately 12 h of sunlight, with two distinct seasons—a dry season and a rainy season—which are not very pronounced.

Sixty fruits were randomly selected for each ripeness stage [11]. Stage M0% corresponded to agronomic maturity, M50% to an intermediate stage characterised by the development of 50% of the fruit’s final colour, and M100% to full maturity, equivalent to 100% of the final colour. Samples were collected from 30 plants located in the inner rows of the plot, excluding those at the edges to minimise potential edge effects on the crop. To standardise the sampling position on the plant, fruits were harvested from the first clusters and selected based on the visual characteristics defined for each ripeness stage (M0%, M50%, and M100%). All fruits corresponding to the stages of ripeness assessed were harvested during a single sampling session.

#### Physicochemical Analysis

Of the selected fruits, 30 were used for physicochemical analysis. For whole fruits, weight, equatorial diameter and longitudinal diameter were determined. For the analysis of pH, soluble solids, and titratable acidity, the tomatoes were cut and homogenised, and the resulting juice was used for the corresponding determinations. pH was measured using a SevenMulti S47 potentiometer (Mettler Toledo, Columbus, OH, USA) [12], whilst soluble solids were determined as Brix using a Hitech RHB-32 ATC manual refractometer (G-Won Hitech Co., Ltd., Seoul, Republic of Korea) [13]. Titratable acidity was determined by weighing 1 g of homogenised sample, which was subsequently diluted in 10 mL of distilled water. The solution was titrated with 0.1 N NaOH using 0.1% phenolphthalein as an indicator.

To determine the moisture content, 1 g of ground sample was weighed and dried in a Memmert BE20 oven (Memmert GmbH + Co. KG, Schwabach, Germany) at 121 °C until a constant weight was reached. The ash content was determined by calcining 1 g of ground sample in a Thermolyne muffle furnace (Thermo Fisher Scientific, Waltham, MA, USA) at 550 °C until white ash was obtained.

The remaining 30 fruits were cut into small pieces, frozen at −80 °C and subsequently freeze-dried using a Christ Alpha 1-4 LDplus unit (Martin Christ Gefriertrocknungsanlagen GmbH, Osterode am Harz, Germany). The freeze-dried material was ground to a fine, homogeneous powder and stored in amber bottles protected from light until the relevant analyses were carried out.

### 2.2. Determination of Minerals

The minerals were extracted in triplicate from 40 mg of freeze-dried tomato powder, to which 5 mL of 65% nitric acid was added. The mixture was subjected to microwave-assisted digestion in a Speedwave Xpert unit (Berghof Products + Instruments GmbH, Eningen unter Achalm, Germany) using a digestion programme involving heating to 140 °C, 30 bar and 70% power for 5 min; then to 200 °C, 35 bar and 80% power for 15 min; and finally cooling to 50 °C and 25 bar, without applying power, for 10 min. Once digestion was complete, the resulting solution was recovered and made up to 25 mL with deionised water. The digests were stored in amber glass vials until analysis [14].

The concentrations of calcium (Ca), iron (Fe), potassium (K), magnesium (Mg) and sodium (Na) were determined by atomic absorption spectroscopy using a Varian SpectrAA 55 instrument (Agilent Technologies, Santa Clara, CA, USA). The wavelengths used were 422.7 nm for Ca, 372.0 nm for Fe, 589.6 nm for Na, 404.4 nm for K and 202.6 nm for Mg. Quantification was performed using calibration curves prepared from 1000 ppm standard solutions, covering a concentration range of 0–200 ppm, prepared with deionised water. All samples were analysed in duplicate, and the results were expressed as mg/100 g dry weight.

### 2.3. Determination of Bioactive Compounds

The analysis of bioactive compounds included quantification of vitamin C, organic acids (citric, malic, and tartaric), carotenoids, chlorophyll and derivatives, and phenolic compounds. For each analysis, 20 mg of freeze-dried powder and a specific solvent were used, depending on the compound of interest. The samples were homogenised in a vortex mixer (Interbiolab Inc., Orlando, FL, USA), subjected to ultrasound-assisted extraction in an ultrasonic bath (Fisher Scientific Inc., Waltham, MA, USA) at 25 °C and 40 kHz and a time that depended on the analysis, and subsequently centrifuged in a microcentrifuge (Eppendorf AG, Hamburg, Germany) at 14,000 rpm, 4 °C for 5 min. The supernatant was recovered and filtered through a 0.45 µm PVDF membrane. Quantification was performed using rapid resolution liquid chromatography (RRLC 1200; Agilent Technologies, Mississauga, ON, Canada), equipped with a DAD-UV-Vis detector. The mobile phase was pumped at a flow rate of 1 mL/min, and the chromatograms were processed using Lab ChemStation software version 2.15.26 (Agilent Technologies, Santa Clara, CA, USA). Compound identification was carried out by comparing retention times and UV-Vis spectra with those of the corresponding analytical standards. Extraction and quantification were performed in duplicate. All standards were prepared at a concentration of 1 mg/mL. The results were expressed as mg/100 g dry weight [15].

#### 2.3.1. Vitamin C

Vitamin C was extracted using a mixture of 1200 µL of 3% metaphosphoric acid and 200 µL of 0.2% *DL*-homocysteine. The mixture was homogenised, shaken for 1 min and then made up to 2 mL with deionised water. Following centrifugation and filtration, 20 µL of the extract was analysed at 244 nm using a Zorbax Eclipse XDB-C18 column (80 Å, 4.6 mm × 50 mm, 1.8 µm) (Agilent Technologies, Santa Clara, CA, USA). The mobile phase consisted of 1.5% potassium monobasic phosphate and 1.8% n-acetyl-n,n,n-trimethylammonium bromide (90:10) pumped at a constant flow rate of 1 mL/min. Quantification was performed using *L*-(+)-ascorbic acid as a standard. Serial injections ranging from 1 to 20 µL were used to fit a linear regression model (R^2^ of 0.99). The method exhibited limits of detection (LODs) and quantification (LOQs) of 0.20 ppm and 0.65 ppm, respectively.

#### 2.3.2. Organic Acids

The organic acids were extracted with 1500 µL of 0.02 N sulphuric acid containing 0.02% *DL*-homocysteine and 0.05% metaphosphoric acid. The mixture was shaken and sonicated for 3 min, after which 500 µL of deionised water was added. After centrifugation and filtration, 20 µL of the extract was analysed at 210 nm using a YMC-Triart C18 column (120 Å, 150 mm × 4.6 mm, 3 µm) (YMC Europe GmbH, Dinslaken, Germany). The mobile phase consisted of 0.027% sulphuric acid in water, pumped at a constant flow rate of 1 mL/min. Quantification was performed using *L*-(+)-tartaric, citric, and malic acids from 100 mg/mL stock solutions. Serial injections ranging from 1 to 20 µL were used to fit a linear regression model (R^2^ of 0.99). The method exhibited LODs and LOQs of 0.06 ppm and 0.17 ppm for tartaric acid, 0.13 ppm and 0.39 ppm for malic acid, and 0.08 ppm and 0.26 ppm for citric acid, respectively.

#### 2.3.3. Carotenoids, Chlorophyll and Derivatives

The carotenoids, chlorophyll, and derivatives were extracted using a mixture of 250 µL methanol, 500 µL chloroform, and 250 µL of deionised water. The mixture was shaken and sonicated for 2 min. The coloured phase was recovered, and the extraction procedure was repeated until the solid residue had lost all colour. The combined extracts were concentrated to dryness in a Buchi R-100 rotary evaporator (Fisher Scientific, Hampton, NH, USA) at a temperature below 30 °C [16]. The dry residue was resuspended in 40 µL of ethyl acetate, and 20 µL of the extract was analysed between 350 and 450 nm using a YMC C30 column (4.6 mm × 150 mm, 3 µm) (YMC Europe GmbH, Dinslaken, Germany). Chromatographic separation was performed using a linear gradient of methanol (A), methyl tert-butyl ether (B) and water (C), as follows: 95% A + 5% B + 0% C at 0 min; 95% A + 5% B + 0% C at 5 min; 95% A + 5% B + 0% C at 5 min; 89% A + 11% B + 10% C at 10 min; 89% A + 11% B + 0% C at 10 min; 75% A + 25% B + 0% C at 16 min; 40% A + 60% B + 0% C at 20 min; 15% A + 85% B + 0% C at 22 min; 90% A + 5% B + 5% C at 25 min; and 90% A + 5% B + 5% C at 28 min, pumped at a constant flow rate of 1 mL/min. The standards used were astaxanthin, violaxanthin, lutein, zeinoxanthin, zeaxanthin, trans-β-apo-8-carotenal, α-carotene, β-carotene, β-cryptoxanthin, lycopene, chlorophyll a, pheophytin a, and pheophytin b. Serial injections ranging from 1 to 20 µL were used to fit a linear regression model (R^2^ of 0.99). LOD and LOQ values for the most important carotenoids were 0.007 and 0.02 ppm for lutein, 0.029 and 0.09 ppm for β-carotene, and 0.003 and 0.008 ppm for zeaxanthin, respectively.

#### 2.3.4. Phenolic Compounds

The phenolic compounds were extracted using 1 mL of 80% methanol acidified with 0.1% HCl. The mixture was shaken and sonicated for 3 min. Following centrifugation, the supernatant was collected, and the solid residue was re-extracted twice more with 500 µL of the same extracting solution. The extracts were combined, filtered, and 20 µL of extracts was analysed between 280 and 370 nm using a Zorbax Eclipse Plus C18 column (4.6 mm × 150 mm, 5 µm) (Agilent Technologies, Santa Clara, CA, USA). The mobile phase consisted of a linear gradient of 0.01% formic acid (A) and acetonitrile (B), as follows: 100% at 0 min, 95% A and 5% B at 5 min, and 50% A and 50% B at 20 min, followed by column washing and re-equilibration, pumped at a constant flow rate of 1 mL/min. The standards used were syringic acid, vanillic acid, ferulic acid, gallic acid, caffeic acid, chlorogenic acid, *m*-coumaric acid, *o*-coumaric acid, *p*-coumaric acid, *p*-hydroxybenzoic acid, chrysin, 2,5-dihydroxybenzoic acid, naringin, quercetin, shikimic acid, 3-hydroxybenzoic acid, kaempferol and luteolin. Serial injections ranging from 1 to 20 µL were used to fit a linear regression model (R^2^ of 0.99). LOD and LOQ for the most important phenolics were 0.009 ppm and 0.028 ppm for chlorogenic acid, 0.048 ppm and 0.145 ppm for caffeic acid, and 0.007 ppm and 0.021 ppm for gallic acid, respectively.

#### 2.3.5. Total Anthocyanin

The total anthocyanin content was determined using the pH differential method. To this end, the extract was prepared by mixing 20 mg of freeze-dried tomato powder with 2 mL of absolute ethanol. The mixture was homogenised and sonicated for 3 min. It was then centrifuged, and the supernatant was recovered for analysis. To determine the anthocyanin content, 50 µL of the extract was placed in a microplate and mixed with 200 µL of 0.025 M potassium chloride buffer adjusted to pH 1.0. In parallel, 50 µL of the extract was mixed with 200 µL of 0.4 M sodium acetate buffer adjusted to pH 4.5. The absorbances of both solutions were recorded at 520 and 700 nm using a spectrophotometer equipped with a microplate reader. Quantification was performed using a calibration curve prepared with delphinidin chloride at a concentration of 1 mg/mL. The calibration curve was constructed over a concentration range of 0.05 to 0.2 mg/mL, with an R^2^ of 0.99. The results were expressed as mg of delphinidin chloride per 100 g of dry weight [17].

### 2.4. Determination of Antioxidant Activity

Antioxidant activity was assessed using the ABTS and DPPH methods. For both assays, the extract was prepared by mixing 20 mg of freeze-dried tomato powder with 2 mL of methanol. The mixture was shaken and sonicated for 3 min. It was then centrifuged at 14,000 rpm and 4 °C for 5 min. The supernatant was recovered and filtered through a 0.45 µm PVDF membrane. Quantification was performed using calibration curves prepared with Trolox dissolved in ethanol over the concentration range of 0.1–4 mM, with an R^2^ of 0.99. The results were expressed as mmol Trolox equivalents per 100 g dry weight.

For the ABTS assay, the cationic radical was prepared by mixing equal volumes of 7 mM ABTS and 2.45 mM potassium persulfate. The solution was incubated in the dark for 16 h to allow radical formation and stabilisation. Subsequently, the absorbance was adjusted to 0.70 by dilution and measured at 734 nm. The reaction was carried out by mixing 20 µL of the extract with 280 µL of the ABTS solution and 300 µL of methanol as a blank. The mixture was shaken, incubated for 15 min, and absorbance was measured using a BioTek H1 microplate reader (Agilent Scientific Instruments, Santa Clara, CA, USA).

For the DPPH assay, the radical was prepared at a concentration of 0.2 mg/mL in methanol. The reaction involved mixing 20 µL of the extract with 280 µL of the DPPH solution and 300 µL of methanol as a blank. The mixture was shaken, allowed to react for 40 min, and the absorbance was measured at 515 nm.

### 2.5. Determination of Antimicrobial Activity

Previous studies on tomato have shown that bioactive compounds segregate during the early stages of ripening and in fully ripe fruit [7,8,16]; therefore, extracts were selected at the M0% stage, corresponding to agronomic maturity, and at the M100% stage, corresponding to full maturity.

The extract used to assess antimicrobial activity was prepared from 2.5 g of freeze-dried tomato powder, which was mixed with 25 mL of 50% ethanol. The mixture was homogenised and subjected to ultrasound-assisted extraction for 6 min. It was then centrifuged at 7500 rpm and 4 °C for 5 min using a centrifuge (Eppendorf, Bochum, Germany). The supernatant was recovered, and the solid residue was re-extracted twice more with the same volume of hydroalcoholic solution. The supernatants obtained were combined and filtered through Whatman No. 1 filter paper. Subsequently, the extract was concentrated by evaporation in a rotary evaporator at a temperature below 40 °C. The resulting concentrate was frozen at −80 °C and subsequently freeze-dried. The dry ethanolic extract obtained was recovered and stored for subsequent use in antibacterial assays (*Escherichia coli* ATCC 8739, *Staphylococcus aureus* ATCC 6538P, *Pseudomonas aeruginosa* ATCC 9027 and *Streptococcus mutans* ATCC 25175) and antifungal assays (*Candida albicans* ATCC 1031 and *Candida tropicalis* ATCC 13803).

The antimicrobial activity of the extracts was evaluated using the broth microdilution method in sterile 96-well microplates. Lyophilised extracts were used to prepare stock solutions by weighing 300 mg of each sample and dissolving them in 5% dimethyl sulfoxide (DMSO). Serial two-fold dilutions were then prepared directly in the microplate.

For the antibacterial assay, 100 µL of sterile Brain Heart Infusion broth (BHI) was added to each well. Subsequently, 100 µL of the extract stock solution was added to the first column and serially diluted by transferring 100 µL from one well to the next, with careful mixing between each transfer. To maintain a constant final volume across wells, 100 µL was discarded from the penultimate dilution column. After the serial dilution of the extracts, 20 µL of a bacterial inoculum was added to each well. The bacterial suspension was initially adjusted to the 0.5 McFarland standard, corresponding to approximately 1–2 × 10^8^ CFU/mL, and subsequently diluted in sterile broth to obtain a final inoculum of approximately 5 × 10^5^ CFU/mL per well, as recommended for broth microdilution assays. The final volume in each well was 120 µL.

For the antifungal assay, the same microdilution procedure was followed using sterile Yeast Extract Peptone Dextrose broth (YPD) as the culture medium. Yeast inocula were standardised to approximately 1.5 × 10^6^ CFU/mL before inoculation. A 20 µL volume of the yeast suspension was added to each well, resulting in a final volume of 120 µL per well.

In both assays, appropriate controls were included in the microplates. Ciprofoxacin was used as a positive control for bacterial strains (stock solution of 1560 µg/mL), and fluconazole was used as a positive control for *Candida* spp. at (stock solution of 1250 µg/mL). The control wells included: a positive control consisting of culture medium, microbial inoculum, and antimicrobial agent; a growth control containing culture medium and microbial inoculum without extract; and a negative control containing only sterile culture medium. The microplates were incubated at 37 °C for 24 h.

Microbial growth was evaluated using a colourimetric viability assay with 2,3,5-triphenyltetrazolium chloride (TTC). After the incubation period, 20 µL of TTC solution was added to each well, and the plates were incubated again at 37 °C for 1–2 h in the dark. TTC was used as a redox indicator because metabolically active, viable cells reduce it, producing a visible red colouration. Wells without visible colour change were interpreted as showing inhibition of microbial growth.

For *Candida* spp., an additional growth confirmation step was performed on Sabouraud Dextrose Agar (SDA). After 24 h of microplate incubation, 4 µL from each well was transferred onto previously labelled and gridded SDA plates. The plates were incubated at 37 °C for an additional 24 h, and the presence or absence of fungal growth was recorded. This procedure confirmed yeast viability after a total incubation period of 48 h. All assays were performed in triplicate.

### 2.6. Statistical Analysis of the Variables Under Study

The statistical analysis was carried out using RStudio (version 4.4.1) and Statgraphics Centurion XVII software. The results are expressed as mean ± standard deviation. Differences between treatments were assessed using analysis of variance (ANOVA), and means were compared using the appropriate test at the 95% confidence level (*p* < 0.05). In addition, a principal component analysis (PCA) was performed to identify clustering patterns and associations between the variables evaluated. Furthermore, a Pearson correlation matrix was constructed at the 95% confidence level (*p* < 0.05) to visualise the strength and direction of the relationships among the physicochemical parameters, minerals, bioactive compounds, antioxidant activity, and antimicrobial activity.

## 3. Results

### 3.1. Market Quality

Table 1 presents the commercial quality characteristics of the five tomato accessions evaluated at three stages of ripeness, including weight, size, pH, soluble solids, total titratable acidity, moisture content, and ash content. The tomato cherry ‘Heart’ recorded the highest values for weight, longitudinal diameter and equatorial diameter compared to the other accessions. Furthermore, a trend towards increased weight was observed as the fruits ripened.

The pH values indicated that all accessions were slightly acidic regardless of their stage of ripeness. Soluble solids content ranged from 4.7 to 7.3 °Brix, with no consistent trend associated with ripening. Similarly, total titratable acidity was highest in the ‘Yellow oval’ and ‘Round green’ cherry tomatoes, although no clear pattern of variation was observed across the different stages of ripeness. Moisture content ranged from 83.0% to 93.8%, whilst ash content ranged from 0.7% to 2.5%. For both parameters, no consistent trend related to the degree of ripeness was identified. Furthermore, the statistical analysis comparing the accessions at the same stage of ripeness revealed significant differences in all cases.

### 3.2. Minerals Profile

Table 2 presents the mineral profile of the tomato accessions assessed at different stages of ripeness. The analysis included quantification of sodium (Na), macronutrients such as calcium (Ca), potassium (K), and magnesium (Mg), and micronutrients such as iron (Fe).

Magnesium was found in the highest concentrations in the ‘Round green’ and ‘Round black’ cherry tomatoes. In general, this mineral tended to increase at the M100% stage; however, this behaviour was not observed in the ‘Round black’ and ‘Heart’ cherry tomatoes. Iron, meanwhile, was not detected at the M0% stage in the ‘Yellow oval’, ‘Red oval’ and ‘Heart’ cherry tomatoes. However, in most accessions, the highest concentrations of this micronutrient were recorded at the M50% stage, except in ‘Red oval’ cherry tomatoes.

Sodium concentrations were highest in the ‘Red oval’ and ‘Heart’ cherry tomatoes. In the latter, its content decreased progressively as the ripening process progressed. Finally, statistical analysis conducted across accessions at the same stage of ripeness revealed significant differences for all minerals evaluated.

### 3.3. Bioactive Compounds

Table 3 shows the concentrations of bioactive compounds, including vitamin C, organic acids (citric, malic, and tartaric), total organic acid content, and total anthocyanins, across the different accessions and stages of ripeness evaluated.

Vitamin C content showed an upward trend as ripening progressed in the ‘Round black’, ‘Red oval’ and ‘Heart’ cherry tomatoes. As for organic acids, citric acid was the predominant compound, followed by malic acid. Citric acid content increased as ripening progressed in the ‘Red oval’ and ‘Heart’ cherry tomatoes, whilst a decrease in concentration was observed in ‘Round green’. Malic acid was found in the highest concentrations in the ‘Yellow oval’ and ‘Red oval’ cherry tomatoes. Furthermore, this compound decreased with ripening in ‘Yellow oval’, whilst it showed an increasing trend in ‘Round black’ and ‘Red oval’. In the case of tartaric acid, the concentration decreased as ripening progressed in the ‘Heart’ cherry tomatoes. Total organic acids reached their highest concentrations in the ‘Yellow oval’ cherry tomatoes. Furthermore, the total content of these compounds increased with ripening in ‘Round black’, whilst it decreased in ‘Round green’.

On the other hand, total anthocyanins were found in the highest concentrations in the ‘Round black’ cherry tomatoes. In these same accessions, the anthocyanin content increased progressively as the fruit ripened. Finally, statistical analysis comparing accessions at the same stage of ripeness revealed significant differences across all evaluated bioactive compounds.

Table 4 presents profiles of carotenoids, chlorophylls, and their derivatives across tomato accessions evaluated by ripeness stage. Among the carotenoids identified, the major compounds were α-carotene, β-carotene, lutein, zeaxanthin, β-cryptoxanthin and lycopene.

In most accessions, the highest concentrations of carotenoids were observed at the M100% ripeness stage, with lycopene as the predominant compound, followed by β-carotene. This trend was observed across most of the materials evaluated, except for the ‘Round green’ cherry tomatoes of β-carotene and the ‘Yellow oval’ and ‘Round green’ cherry tomatoes of lycopene. This behaviour is consistent with the characteristic green colouration of the ‘Round green’ cherry tomatoes. Lutein was present in all maturity stages and accessions analysed; however, no defined pattern of variation associated with the ripening process was observed. Similarly, the total carotenoid content tended to reach its highest values at M100%, except in the ‘Round green’ cherry tomatoes, where this behaviour was not evident.

Regarding the profile of chlorophylls and their derivatives, the predominant compounds were pheophytin b and chlorophyll b. The highest concentration of pheophytin b was recorded at the M0% stage for all accessions, except in ‘Round black’, where the maximum value was observed at M50%. Chlorophyll b, meanwhile, was detected mainly in the ‘Yellow oval’ and ‘Round green’ cherry tomatoes. Finally, statistical analysis across accessions at the same maturity stage revealed significant differences in most evaluated carotenoids, chlorophylls, and derivatives.

Table 5 presents the phenolic compound profile of the tomato accessions evaluated at different stages of ripeness. The predominant phenolic compounds were gallic acid, 4-hydroxybenzoic acid, *m*-coumaric acid, syringic acid, chlorogenic acid, naringin, caffeic acid, ferulic acid, kaempferol, quercetin glucoside and quercetin.

In general, no single predominant phenolic compound was identified across all accessions and stages of ripeness; instead, accumulation profiles varied by accessions and ripeness stage. Gallic acid, 4-hydroxybenzoic acid, and syringic acid were present at all evaluated stages of ripeness. The gallic acid content increased with ripening in the ‘Round black’ and ‘Red oval’ cherry tomatoes, whilst it decreased in the ‘Round green’ cherry tomatoes.

4-Hydroxybenzoic acid showed an increasing trend with ripening in the ‘Round black’, ‘Red oval’ and ‘Heart’ cherry tomatoes. Similarly, syringic acid increased in ‘Yellow oval’, ‘Round green’ and ‘Heart’, whilst it decreased in ‘Round black’. On the other hand, chlorogenic acid showed a progressive decrease in the ‘Heart’ cherry tomatoes as ripening progressed.

Caffeic acid content decreased with increasing ripeness in the ‘Round green’ cherry tomatoes. In the case of kaempferol, a reduction in its concentration was observed in ‘Yellow oval’, whilst in ‘Round black’ and ‘Heart’ an increase associated with the ripening process was recorded. Quercetin glucoside showed an upward trend in ‘Round black’ and a downward trend in ‘Heart’. A similar pattern was observed for quercetin, with its concentration increasing in ‘Round black’ and decreasing in ‘Heart’ as ripening progressed. Finally, the total phenolic content, calculated as the sum of the identified individual phenolic compounds, increased with ripeness in the ‘Heart’ cherry tomatoes.

### 3.4. Antioxidant Activity

Table 6 presents the antioxidant activity results for the tomato accessions assessed at different stages of ripeness using the DPPH and ABTS methods. The antioxidant activity values determined by the DPPH method ranged from 1.2 to 4.7 mmol Trolox equivalents per 100 g dry weight (mmol TE/100 g DW), whilst those obtained using the ABTS method ranged from 1.5 to 3.6 mmol TE/100 g DW.

In most accessions, the antioxidant activity measured by the DPPH method reached its highest value at the M100% ripeness stage, except in the ‘Round green’ cherry tomatoes. A similar pattern was observed for the ABTS method, with the highest antioxidant activity occurring at M100%, except for the ‘Round green’ cherry tomatoes. Finally, statistical analysis conducted across accessions within the same ripeness stage revealed significant differences in antioxidant activity as determined by both methods.

### 3.5. Antimicrobial Activity

Table 7 shows the minimum inhibitory concentration (MIC) values for the freeze-dried ethanolic extracts obtained from the different tomato accessions at 0% and 100% ripeness. In general, the extracts exhibited moderate antibacterial activity against the strains tested, except for the extract from the ‘Heart’ cherry tomatoes, which showed no activity against *Staphylococcus aureus*, *Pseudomonas aeruginosa*, and *Streptococcus mutans*.

For *Escherichia coli*, the MIC values ranged from 5.21 to 31.46 mg/mL. In the case of *Staphylococcus aureus*, the minimum inhibitory concentrations ranged from 13.35 to 83.96 mg/mL, whilst for *Pseudomonas aeruginosa*, they ranged from 26.71 to 83.96 mg/mL. *Streptococcus mutans*, meanwhile, showed the widest range of variation, with inhibitory concentrations ranging from 1.95 to 83.75 mg/mL.

In contrast, none of the extracts evaluated showed antifungal activity against *Candida albicans* and *Candida tropicalis* under the experimental conditions used. Finally, the results demonstrate greater sensitivity of the bacteria evaluated compared to the yeasts, which showed no growth inhibition within the concentration range analysed.

### 3.6. Statistical Analysis

**Figure 2:**
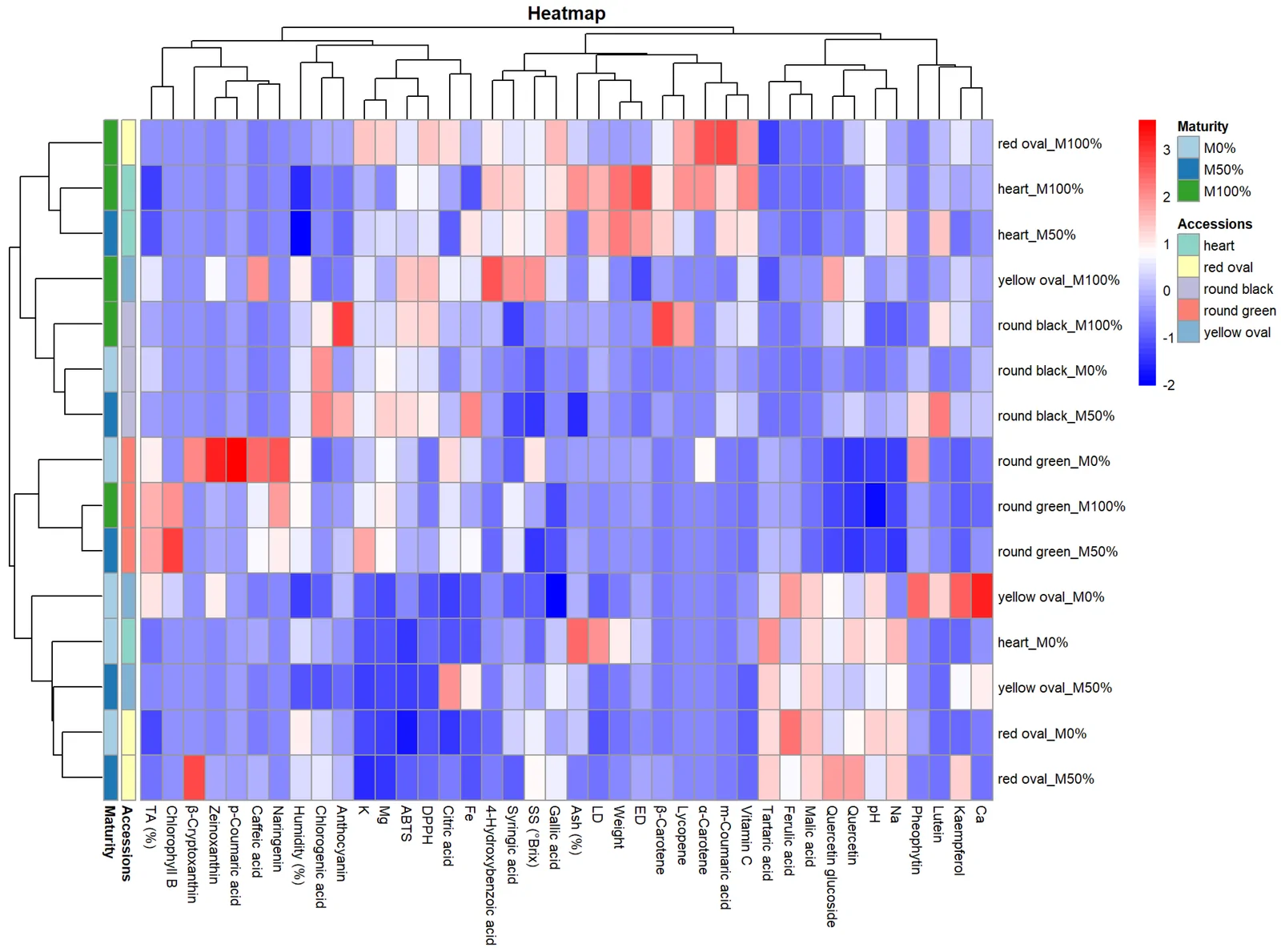
Hierarchical cluster analysis of five cherry tomato accessions at three stages of ripeness, taking into account the variables under study. Note: TA, Titratable acidity; K, Potassium; Mg, Magnesium; ATS, Antioxidant activity by ABTS; DPPH, Antioxidant activity by DPPH; Fe, iron; SS, Soluble solids; LD, longitudinal diameter; ED, Equatorial diameter; Na, sodium; Ca, calcium.

Figure 2 shows the heat map from a hierarchical clustering analysis of tomato accessions across three ripeness stages (M0%, M50%, and M100%), based on physicochemical variables, minerals, carotenoids, chlorophylls, organic acids, phenolic compounds, and antioxidant capacity. The dendrogram shows that the variables are grouped primarily by accessions rather than by ripeness stage, suggesting that accessions have a greater influence on chemical composition than ripeness does.

**Figure 3:**
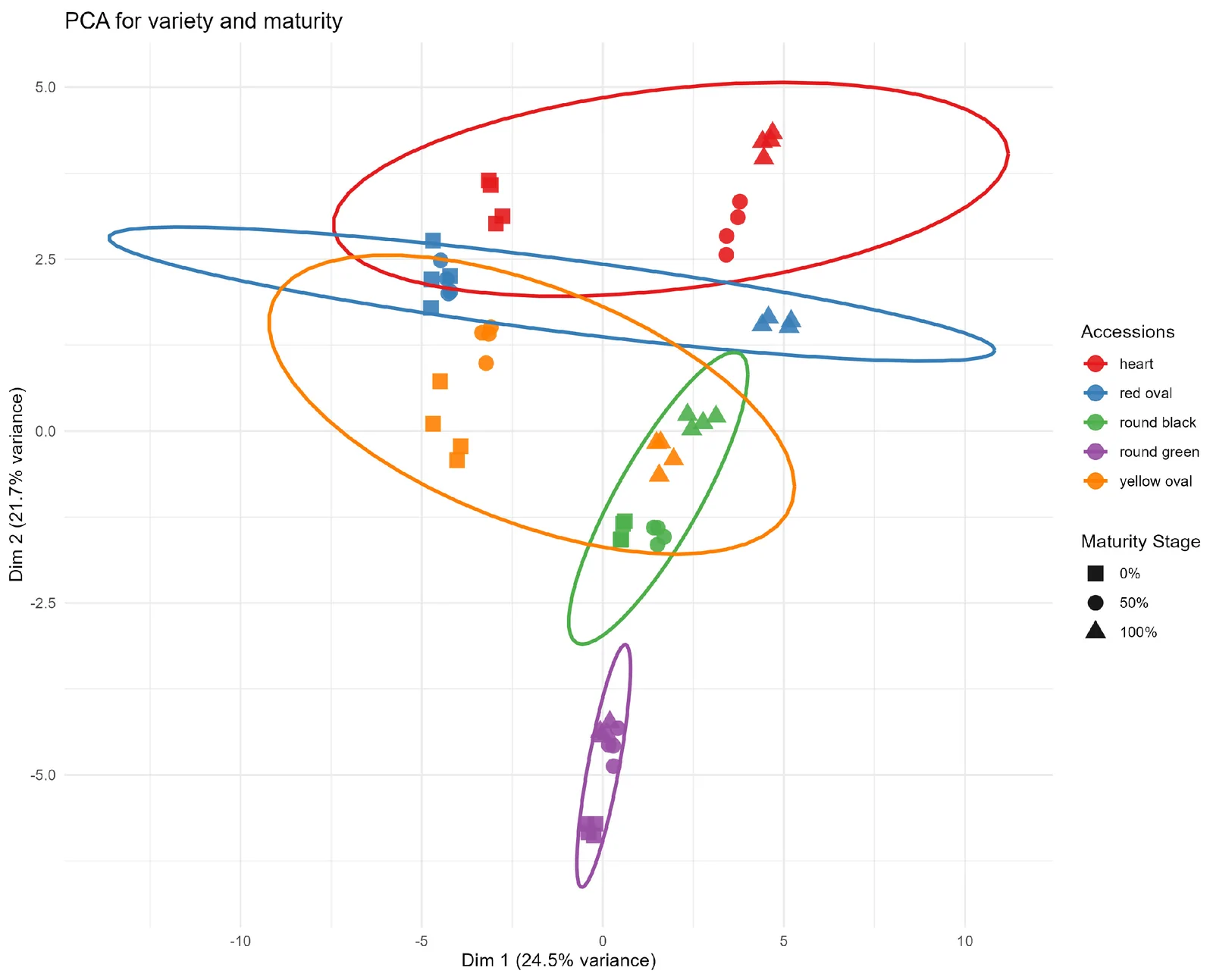
Principal component analysis taking into account accessions and stage of ripeness.

Figure 3 presents the principal component analysis (PCA), focusing primarily on the accessions under study and their maturity stages. The PCA explains 46.2% of the total variability, with Dimension 1 (Dim1) accounting for 24.5% and Dimension 2 (Dim2) for 21.7%. Although the cumulative percentage is moderate, it is sufficient to identify biologically relevant patterns associated with accessions and ripeness stage.

**Figure 4:**
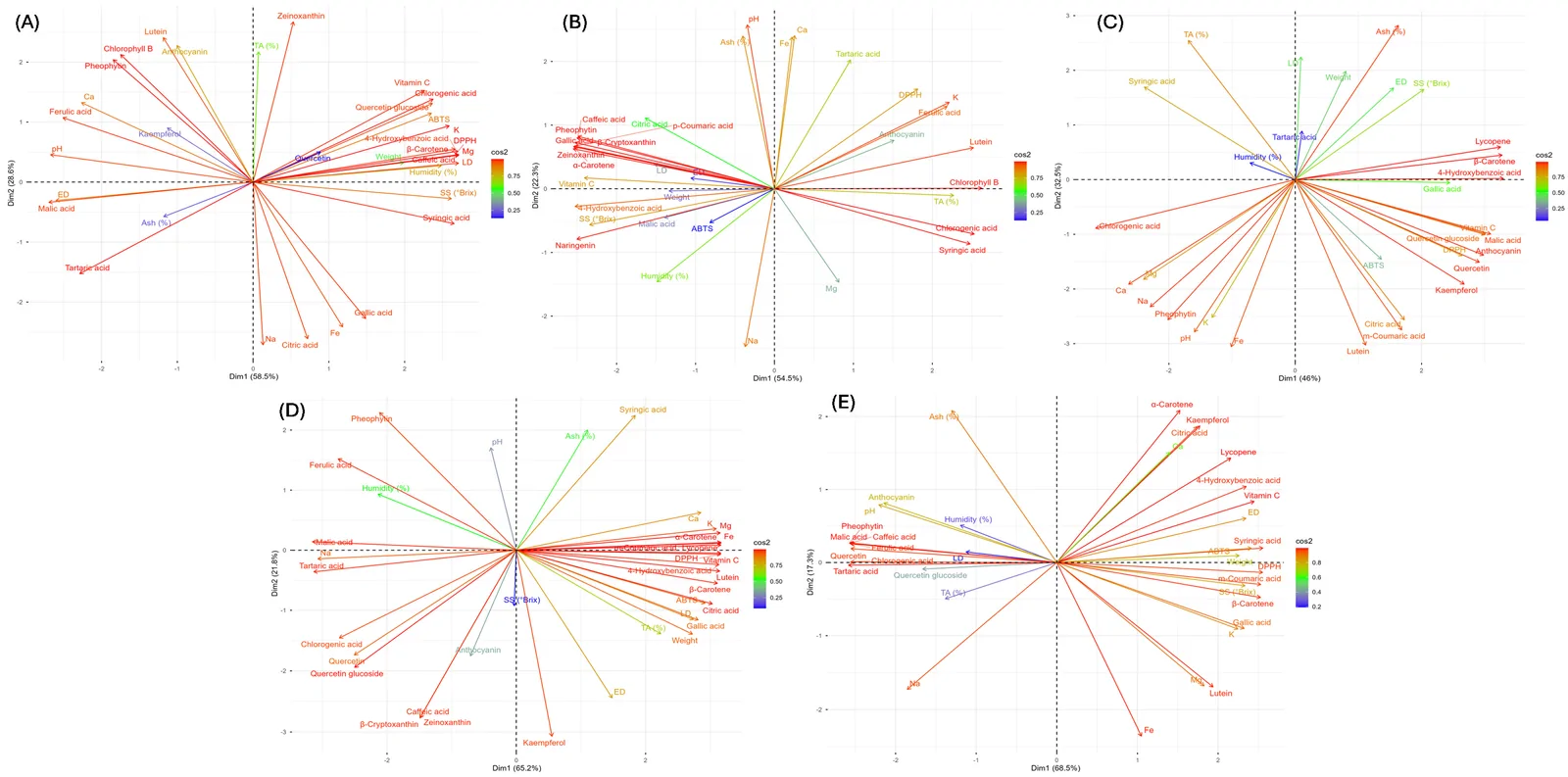
Principal component analysis (PCA) for the accessions under study. (**A**) ‘Yellow oval’ PCA; (**B**) ‘Round green’ PCA; (**C**) ‘Round black’ PCA; (**D**) ‘Red oval’ PCA; (**E**) ‘Heart’ PCA. Note: TA, Titratable acidity; K, Potassium; Mg, Magnesium; ATS, Antioxidant activity by ABTS; DPPH, Antioxidant activity by DPPH; Fe, iron; SS, Soluble solids; LD, longitudinal diameter; ED, Equatorial diameter; Na, sodium; Ca, calcium.

Figure 4 presents the principal component analysis for the accessions under study. The two dimensions accounted for between 76.8 and 87.1 of the total variability, with Dim1 ranging from 46.0 to 68.5 per cent and Dim2 from 17.3 to 32.5 per cent.

In Figure 4A, on Dim1, a positive cluster is observed for lycopene, β-carotene, vitamin C, ABTS, DPPH, weight, longitudinal diameter, and soluble solids, indicating that these variables collectively contribute to the differentiation of the samples. The angular proximity between lycopene and β-carotene demonstrates a positive association, consistent with the biosynthetic pathway of carotenoids. Furthermore, the similar orientation of ABTS and DPPH relative to vitamin C and carotenoids suggests that antioxidant activity is associated with the accumulation of these bioactive compounds. In contrast, chlorophyll a, chlorophyll b, lutein, and anthocyanins are located in the opposite quadrant, showing an inverse relationship with the major carotenoids.

Figure 4B shows that lycopene, β-carotene, lutein, potassium and ferulic acid have vectors oriented in the same positive direction as Dim1, suggesting an association between these metabolites. The proximity of lycopene and β-carotene once again highlights a coordinated behaviour of the carotenoids during ripening. Similarly, ABTS is located close to magnesium and moisture, whilst DPPH appears associated with potassium and ferulic acid. The arrangement of the vectors suggests that antioxidant activity may be related to phenolic metabolites and carotenoid pigments present in the samples. Furthermore, vitamin C, α-carotene, zeaxanthin, gallic acid and caffeic acid are situated in the quadrant opposite to lycopene and β-carotene, indicating differential patterns of accumulation amongst groups of bioactive compounds.

Figure 4C shows a very marked positive association between lycopene, β-carotene, vitamin C, anthocyanins, quercetin, quercetin glucoside and malic acid, whose vectors are oriented in the same positive region of Dim1. The proximity of these compounds suggests that they jointly contribute to the differentiation of the samples. ABTS is located close to this group, which is consistent with evidence linking increased antioxidant activity to the accumulation of carotenoids and polyphenols during ripening. In contrast, chlorogenic acid, pH, pheophytin, iron and sodium are situated in the opposite direction, indicating a different behaviour compared with the set of metabolites associated with physiological ripeness.

In Figure 4D, Dim1 accounts for most of the variation and is dominated by lycopene, β-carotene, vitamin C, DPPH, ABTS, weight, potassium and magnesium, all oriented in the same positive direction. The proximity of DPPH, ABTS, and vitamin C indicates a consistent association between antioxidant capacity and the bioactive compounds present in the samples. Conversely, chlorogenic acid, quercetin glucoside, caffeic acid and zeaxanthin are located in the negative direction of Dim2, suggesting that these variables explain a distinct fraction of the variability. In Figure 4E, PCA exhibits the greatest explanatory power of the five analyses. The main multivariate structure is defined by lycopene, vitamin C, α-carotene, kaempferol, citric acid, weight, DPPH, β-carotene and potassium, whose vectors show a very similar orientation. This configuration indicates that these compounds collectively contribute to the differentiation of the samples. ABTS is closely related to DPPH, confirming consistency between the two antioxidant assays. Furthermore, pH, anthocyanins, caffeic acid, malic acid, tartaric acid, and chlorophyll A show opposite trends, reflecting distinct metabolic patterns.

In Figure 4E, PCA exhibits the greatest explanatory power of the five analyses. The main multivariate structure is defined by lycopene, vitamin C, α-carotene, kaempferol, citric acid, weight, DPPH, β-carotene and potassium, whose vectors show a very similar orientation. This configuration indicates that these compounds collectively contribute to the differentiation of the samples. ABTS is closely related to DPPH, confirming consistency between the two antioxidant assays. Furthermore, pH, anthocyanins, caffeic acid, malic acid, tartaric acid, and chlorophyll A show opposite trends, reflecting distinct metabolic patterns.

## 4. Discussion

### 4.1. Market Quality

All accessions were cultivated in the same open-field location and were subjected to the same general agronomic and sampling conditions. This common-environment design reduces major environmental differences among accessions and strengthens the comparative interpretation of the results within the experimental field. Nevertheless, plants grown under open-field conditions remain exposed to spatial and temporal variation in solar radiation, temperature, soil properties, water availability, and other microenvironmental factors. Because the study included only one location, one cultivation cycle, and no reference accessions grown in another environment, the effects of Ecuadorian growing conditions cannot be separated from accession effects or accession-by-environment interactions.

The differences observed in weight, longitudinal diameter, and equatorial diameter among the samples studied indicate marked accession-dependent differences in fruit size and morphology. The increase in weight observed in the ‘Red oval’ and ‘Heart’ cherry tomatoes as ripening progressed can be attributed to cell expansion and the progressive accumulation of water and metabolites during fruit development. During the transition from physiological maturity to consumption maturity, the tomato undergoes metabolic changes, including the accumulation of storage compounds and an increase in cell volume [18,19]. In turn, the ‘Heart’ cherry tomatoes exhibited the largest dimensions, whilst ‘Red oval’ recorded the smallest sizes in the early stages of maturity. Other authors have shown that genetic factors primarily determine tomato fruit size, although it can be modulated by environmental conditions and the stage of fruit development [7,20].

Soluble solids are among the main indicators of organoleptic quality due to their close relationship with sugar content and perceived sweetness [3]. In the present study, values ranging from 4.7 to 7.3 °Brix were recorded, with the highest observed in ‘Yellow oval’ at M100%. The results at maturity showed some correlation with yellow and red tomatoes grown under glass in Iran [21]. Furthermore, other authors have reported high concentrations of soluble solids in yellow tomatoes [22]. On the other hand, these results are consistent with those of other authors who reported increases in soluble solids during ripening due to the accumulation of sugars derived from the hydrolysis of complex carbohydrates and the mobilisation of photoassimilates towards the fruit [5]. However, not all samples showed a uniform increase in soluble solids with ripeness. This behaviour suggests that the response is strongly influenced by accessions, consistent with other authors who report high varietal variability in soluble solids content and in the dynamics of sugar accumulation during tomato ripening [3].

Titratable acidity showed relatively low values, ranging from 0.2 to 0.9%, without a consistent pattern of change associated with ripening. Thus, from a nutritional and sensory perspective, the balance between soluble solids and titratable acidity is one of the factors that most contributes to consumer acceptance of the tomato, as it determines the perceived intensity of sweet and sour flavours [3]. Consequently, ‘Yellow oval’ and ‘Heart’ cherry tomatoes, which combine high soluble solids with moderate acidity, could exhibit favourable organoleptic characteristics.

Moisture content was above 80% across all samples, confirming that tomato is an important source of water in the human diet, as reported by other authors, who found moisture content exceeding 90% in tomato accessions at different stages of ripeness grown in Spain [5]. The high moisture content contributes to the fruit’s palatability and nutritional value, although it also increases susceptibility to postharvest deterioration due to high available water activity [2].

Ash content ranged from 0.2 to 2.5%, reflecting differences in mineral accumulation between samples and stages of ripeness. The variations observed suggest that both accessions and the physiological processes associated with ripening influence the mineral composition of the fruit, a relevant aspect given the contribution of minerals to the nutritional and functional quality of the tomato, as noted by other authors who reported an ash content range of 0.22–0.40% in cherry tomato varieties [5].

### 4.2. Mineral Profile

Table 2 shows that the mineral profiles of the tomato accessions were influenced by both accessions and ripeness stage, as all comparisons between accessions within the M0%, M50%, and M100% groups revealed significant differences in Ca, Fe, K, Mg, and Na. Thus, potassium was the predominant mineral in all samples, confirming the importance of this macronutrient in the mineral composition of tomatoes. The literature describes K as one of the most abundant mineral elements in tomatoes; furthermore, the dynamics of Ca, Fe and Mg may vary depending on variety, plant nutrition and the physiological state of the fruit; therefore, micronutrients do not follow a uniform pattern of accumulation during ripening [23,24].

In this regard, the results show that ripening did not affect all minerals in the same way, as some elements increased by 1100% at M50%, others reached peaks at M50%, and others decreased with ripening. This differential response is consistent with studies noting that tomato composition depends on the variety and the environment [23,25].

From a functional perspective, the ‘Round green’, ‘Round black’ and ‘Red oval’ cherry tomatoes stood out for their high K and Mg contents at certain stages of ripeness, whilst ‘Yellow oval’ stood out for its higher Ca content at M0%. These results are similar to those of other authors who reported differences in the concentrations of minerals such as K, Ca, Mg, and Fe [2,26]. Identifying these differences allows for the selection of accessions and ripeness stages with greater nutritional potential for fresh consumption or for the development of functional foods derived from tomatoes [5]. On the other hand, authors who studied tomatoes grown under different systems in Ecuador reported slightly lower Fe concentrations than those observed in this study. This slight variation may be due to the type of cultivation under study, as the authors point out [27].

### 4.3. Bioactive Compounds

Table 3 shows that both accessions and stage of ripeness influenced the composition of bioactive compounds. Comparisons among different accessions at the same ripeness level showed significant differences in vitamin C, organic acids, and anthocyanins. This pattern aligns with observations from other authors, who emphasise that the accumulation of bioactive metabolites in tomatoes is influenced by the interaction between genetic factors and the metabolic changes that occur during ripening [7,8].

Vitamin C exhibited varying behaviour depending on the type of cherry tomato. The ‘Round black’, ‘Red oval’ and ‘Heart’ cherry tomatoes showed a steady increase in ascorbic acid levels as they ripened, reaching their peak concentrations at M100%. This behaviour aligns with findings from other authors who have reported high concentrations of vitamin C in yellow and black varieties [4]. Additionally, the biosynthesis and accumulation of vitamin C typically increase during ripening due to intensified oxidative metabolism and heightened activity in the biosynthetic pathways associated with ascorbic acid [28]. However, accessions like ‘Heart’ and ‘Red oval’ achieved significantly higher values compared to those observed in the early stages of ripeness. On the other hand, ‘Yellow oval’ displayed high vitamin C levels at M0%, followed by a decrease at M50%, and then an increase at M100%. This pattern has been documented by other authors, who noted that during fruit development, vitamin C content often decreases—likely due to dilution effects—before increasing during the ripening phase [29]. The elevated vitamin C concentrations observed in some accessions may be associated with several interacting factors, including accession identity, ripeness stage, crop management, and environmental conditions. Pichincha is located at approximately 2400 m above sea level and has an average temperature of approximately 18 °C; however, the influence of altitude, solar radiation, temperature, and humidity was not evaluated independently in this study. In this context, research has shown that factors such as light intensity, temperature, and air humidity significantly influence fruit composition [30]. Generally, temperatures exceeding 10 °C during the growth period encourage the accumulation of ascorbic acid, phenolic compounds, and carotenoids [29,31]. Additionally, ensuring a balanced nutrient supply is crucial for preventing the degradation of ascorbic acid and promoting its high levels within the plant [21]. It is essential to note that environmental conditions represent only one of many factors influencing high concentrations of vitamin C, as other interrelated elements also play a significant role, as highlighted by various authors [7,8]. From a nutritional standpoint, this is important because tomatoes serve as a key dietary source of vitamin C, which is essential for antioxidant functions, immune support, and protecting cells against oxidative stress [28].

In the study of acids, citric acid emerged as the predominant compound across most tomato accessions. This finding aligns with the typical composition of the fruit, where citric and malic acids play crucial roles in determining its acidity and sensory balance [2,3,29]. Notably, the ‘Red oval’ and ‘Heart’ cherry tomatoes showed an increase in citric acid levels as they ripened, whereas the ‘Round green’ exhibited a gradual decrease. These findings suggest that the processes involved in the synthesis and degradation of organic acids vary among different accessions, a phenomenon commonly observed during the ripening stage of climacteric fruits [2]. Furthermore, the decrease in organic acids during ripening could be linked to their use as respiratory substrate, a process that typically intensifies as the fruit reaches more advanced ripening stages [18]. Research has also indicated variations in the total organic acid concentrations among the tomatoes under study, with some varieties accumulating higher levels of citric acid than others [21].

On the other hand, anthocyanins showed significant differences across the various accessions. The ‘Round black’ consistently showed the highest concentrations at all ripeness stages. This pattern is in line with recent studies on black or purple tomatoes, where anthocyanin accumulation is directly related to the dark pigmentation of the fruit [4,26,32]. Conversely, the ‘Heart’, ‘Yellow oval’, ‘Red oval’, and ‘Round green’ cherry tomatoes exhibited considerably lower anthocyanin concentrations and did not show the increases typically associated with ripening. This observation is supported by the literature, which notes that most commercial tomatoes have limited anthocyanin content owing to genetic constraints on the expression of the related biosynthetic pathways [6].

#### 4.3.1. Carotenoid Profile

Table 4 shows that the composition of carotenoids and chlorophylls was strongly influenced by both accessions and degree of ripeness. The significant differences observed among accessions at the same stage of ripeness confirm that pigment accumulation in tomatoes depends on genetic mechanisms regulating the biosynthesis and degradation of carotenoids and chlorophylls during fruit development. This response has been extensively documented in cherry tomatoes of different colours, in which pigment composition is a main factor influencing the fruit’s visual appearance and nutritional value [2,8,21]. Furthermore, carotenoid concentration and chlorophyll degradation are influenced by light and ambient temperature; in general, mild to moderate light stress can promote pigment accumulation [31]. Furthermore, the tomato grown in Spain had lower total carotenoid concentrations than those recorded for cherry tomato grown in Ecuador [7,33,34]. These differences could be linked to various factors, including sample type, stage of ripeness, and environmental growing conditions. In this context, Ecuador’s geographical location is characterised by a relatively stable photoperiod throughout the year, with around 12 h of daylight; however, the possible influence of these conditions on carotenoid accumulation should be interpreted with caution, as this study did not directly assess the effect of solar radiation, temperature or other environmental variables on their biosynthesis [29,31].

Among the identified carotenoids, lycopene was the predominant compound in the ‘Round black’, ‘Red oval’ and ‘Heart’ cherry tomatoes, reaching high concentrations at M100%. This result is consistent with the literature, which recognises lycopene as the most abundant carotenoid in ripe red and dark-coloured tomatoes due to the activation of the carotenoid biosynthetic pathway during ripening [5,8]. In turn, red, orange, and brownish tomatoes have the highest total carotenoid concentrations, as shown in a study of 15 cherry tomato varieties grown in Sicily [35]. Furthermore, the increase in lycopene with ripeness reflects one of the most characteristic metabolic changes in tomato ripening [31]. During this process, a transition occurs from green chromoplasts to carotenoid-rich chromoplasts, favouring the accumulation of lycopene and the appearance of intense red colouration [5,7]. Thus, among the chlorophylls and their derivatives, pheophytin b was the predominant compound. The highest concentrations were generally recorded in M0%, particularly in ‘Yellow oval’ and ‘Round green’. This behaviour was expected because pheophytin derives from the degradation of chlorophyll during ripening and reflects the physiological state of the fruit’s photosynthetic system [36].

β-carotene was the second most abundant carotenoid and showed high concentrations in ‘Heart’, ‘Red oval’ and ‘Round black’. This finding is consistent with recent research identifying β-carotene as one of the main precursors of vitamin A in tomatoes, whose accumulation typically increases as ripening progresses [2,36].

#### 4.3.2. Phenolic Profile

Table 5 shows that the phenolic profile was determined mainly by the accessions, whilst ripeness modulated the concentration of specific compounds. This combined influence of accessions and ripeness has been widely reported in tomatoes, where the biosynthesis of phenolic acids and flavonoids depends on the genetic regulation of the phenylpropanoid pathway [19]. Furthermore, the tomato grown in Spain had lower total phenolics concentrations than those recorded for cherry tomato grown in Ecuador [7,33,34]. These differences could be linked to various factors, including stage of ripeness and environmental growing conditions. In this context, Ecuador’s geographical location is characterised by a relatively stable photoperiod throughout the year, with around 12 h of daylight; however, the possible influence of these conditions on phenolics accumulation should be interpreted with caution, as this study did not directly assess the effect of solar radiation, temperature or other environmental variables on their biosynthesis [29,31].

Among the identified compounds, phenolic acids represented the predominant fraction of the phenolic profile. 4-Hydroxybenzoic acid reached particularly high concentrations in ‘Yellow oval’, ‘Red oval’ and ‘Heart’ at M100%, whilst chlorogenic acid predominated in ‘Round green’ and ‘Red oval’. These results are consistent with those of other authors who identify hydroxybenzoic and hydroxycinnamic acids as the main phenolic compounds present in tomatoes [10].

Ripening promoted the accumulation of various phenolic compounds in certain accessions. For example, ‘Round black’, ‘Red oval’ and ‘Heart’ showed increases in gallic acid, 4-hydroxybenzoic acid, quercetin and kaempferol as ripening progressed. This behaviour can be attributed to increased activity of the phenylpropanoid pathway during the final stages of fruit development, a process associated with the synthesis of antioxidant metabolites [19].

The total phenolic compound content increased significantly in ‘Yellow oval’, ‘Heart’ and ‘Red oval’ upon reaching M100%, whilst ‘Round green’ showed a decrease compared to M0%. Similar results have been reported in cherry tomatoes, where ripening generally favours the accumulation of total polyphenols and an increase in antioxidant capacity [35]. However, the magnitude of these changes depends on the variety evaluated, environmental conditions, and agronomic conditions [7,18]. A study of 15 tomato varieties of different colours showed that the total phenolic content in the brownish, red, and yellow tomatoes was, to some extent, related to their content [35].

From a nutritional perspective, the ‘Yellow oval’, ‘Red oval’ and ‘Heart’ cherry tomatoes at M100% exhibited the highest total phenolic contents, whilst ‘Round black’ stood out for the accumulation of specific flavonoids such as quercetin and kaempferol. These compounds have been associated with antioxidant and anti-inflammatory properties; therefore, their increase during ripening could enhance the fruit’s functional value [1].

In most accessions, progression towards full maturity was accompanied by greater carotenoid accumulation and a reduction or transformation of chlorophyll-related pigments, consistent with the transition from photosynthetically active immature fruit to the final accession-dependent colour. However, the ‘Round green’ accession followed a different pattern, with several bioactive variables reaching higher values at the earlier stage. Because its mature phenotype remains green, colour-based maturity criteria may not correspond to the same pigment transition observed in red- or yellow-fruited accessions. Chlorophyll retention, reduced carotenoid accumulation, or a different balance among pigment pathways may contribute to this behaviour, although the underlying mechanism was not directly evaluated.

### 4.4. Antioxidant Activity

Table 6 shows that antioxidant activity was influenced by both accessions and degree of ripeness, with significant differences observed between accessions for both assessment methods. This finding is consistent with recent studies indicating that the antioxidant capacity of tomatoes depends mainly on the composition and concentration of bioactive compounds accumulated during ripening [5]. Thus, this study found the highest antioxidant activity in the black and red tomatoes, followed by the yellow tomatoes; these results are partly consistent with other studies, which indicated that red tomatoes have the highest antioxidant activity, followed by yellow and orange tomatoes [32]. Furthermore, the antioxidant activity observed in this study was higher than that reported for 28 native Mexican tomatoes [37]. These differences may be due to the geographical and climatic characteristics of the regions compared, as suggested by other authors [7,38].

In general, ripening increased antioxidant activity in most accessions. The ‘Yellow oval’, ‘Red oval’ and ‘Heart’ cherry tomatoes showed the greatest increases between M0% and M100%, whilst ‘Round black’ exhibited high values at all stages of ripeness. The results obtained using DPPH showed greater differentiation between ripeness stages than those observed using ABTS. This suggests that changes in antioxidant composition during ripening mainly affected compounds with high hydrogen-transfer capacity, particularly carotenoids and some phenolic compounds, as indicated by the results of other authors on coloured tomatoes [4].

The ‘Red oval’ and ‘Heart’ cherry tomatoes showed a marked increase in antioxidant activity at M100%, coinciding with the higher vitamin C contents observed in these accessions. Similarly, ‘Round black’ exhibited high antioxidant activity associated with the accumulation of lycopene, β-carotene and anthocyanins during ripening. These results suggest that the antioxidant activity was due to the combined action of multiple bioactive compounds rather than a single metabolite [2,5].

From a nutritional perspective, the high concentration of lycopene is notable, as this carotenoid exhibits strong antioxidant capacity and has been associated with protective effects against cardiovascular disease, chronic inflammation, and certain cancers [39].

The higher ABTS and DPPH values generally observed at full maturity, except in ‘Round green’, were consistent with broader changes in the chemical profile during ripening. However, these assays measure radical-scavenging capacity in cell-free chemical systems and respond differently to the composition and reactivity of the extract. Therefore, the observed values should not be interpreted as direct evidence of antioxidant effects in biological systems or attributed to a single compound.

### 4.5. Antimicrobial Activity

The selection of the maturity stages M0% (agronomic maturity) and M100% (full maturity) was based on experimentally obtained profiles of bioactive compounds in the evaluated cherry tomato accessions. As shown in Table 3, the highest vitamin C concentrations were predominantly observed at M100%, except for the ‘Round green’ cherry tomato accession s, where the highest value was observed at M0%. Similarly, the highest concentrations of carotenoids (Table 4) and phenolic compounds (Table 5) were identified at M100% for the accessions studied, except for ‘Round green’, which showed its highest concentrations at M0%. Taken together, these results demonstrated a pattern dependent on accessions and ripeness stage, enabling the selection of M0% and M100% as contrasting, representative stages for assessing antimicrobial activity. This behaviour is consistent with previous studies, which have indicated that the accumulation of carotenoids and phenolic compounds during tomato ripening varies with vegetal material and compound; consequently, maximum concentrations may occur in both early stages and fully ripe fruits [7,8,16].

The accession effect must also be interpreted within the cultivation conditions used in this study. International studies have shown that the growing area can alter tomato gene expression and the abundance of phenylpropanoids, carotenoids, sugars and organic acids in a cultivar-dependent manner, while irrigation regime can modify fruit pH, vitamin C, ethylene production and the broader metabolome [40,41]. Likewise, marked differences in phenolics, flavonoids and carotenoids have been reported among black cherry tomato accessions cultivated under the same greenhouse conditions [42]. Accordingly, the present data support reproducible phenotypic differences among accessions under the evaluated Ecuadorian environment, but they do not by themselves separate genetic effects from accessions × environment interactions. Table 7 shows that the antimicrobial activity of the dried ethanolic extracts depended on both the accessions and the stage of ripeness, with a more pronounced response against bacteria than against fungi. In general, the freeze-dried ethanolic extracts exhibited activity against *Escherichia coli*, *Staphylococcus aureus*, *Pseudomonas aeruginosa* and *Streptococcus mutans*, whilst no activity was detected against *Candida albicans* or *Candida tropicalis* under the conditions evaluated. This pattern supports a predominantly antibacterial response in vitro, which should be interpreted with caution because most evidence on phenolic-rich plant extracts is generated under simplified in vitro conditions and may not translate directly to food matrices or in vivo systems [43]. These results differed from those reported by other authors, who observed no antimicrobial activity against *E. coli* in extracts from yellow and red tomato skins, whereas extracts from green tomato skins exhibited activity against this microorganism. Furthermore, the authors did not record any activity against *S. aureus*, in contrast to the results obtained in the present study. On the other hand, both studies reported no activity against *C. albicans* [44].

Among the bacteria evaluated, *S. mutans* was the most sensitive strain, with the lowest MICs recorded in ‘Heart’ M0% (1.95 mg/mL), ‘Red oval’ M0% (2.61 mg/mL) and ‘Round black’ M100% (5.21 mg/mL). This response may be related to the susceptibility of Gram-positive bacteria to phenolic metabolites present in plant extracts, which can affect the cell membrane, bacterial adhesion and biofilm formation. This restrained interpretation is consistent with a recent international study of polyphenol- and carotenoid-rich tomato pomace, which reported only modest antimicrobial activity overall despite the presence of multiple bioactive compounds [43,45].

The stronger response against *S. mutans* may be associated with the phenolic profile of the extracts rather than with total phenolic content alone. ‘Heart’ M0%, despite showing an intermediate total phenolic content, contained phenolic acids and flavonoid derivatives, including gallic acid, 4-hydroxybenzoic acid, chlorogenic acid, caffeic acid, ferulic acid, quercetin glucoside, and quercetin. ‘Red oval’ M0% showed a higher total phenolic content and a particularly high level of ferulic acid, together with chlorogenic acid and quercetin derivatives. Phenolic compounds may affect bacterial cells through membrane disruption, interference with metabolic pathways, metal chelation, enzyme inhibition and nucleic acid damage; however, because crude extracts were used, the inhibitory effect cannot be assigned to a single compound [43].

The relationship between total phenolics and MIC was not linear. ‘Yellow oval’ M100% showed the highest total phenolic content among the samples, but it was not the extract with the strongest antimicrobial activity. Conversely, ‘Heart’ M0% showed the lowest MIC against *S. mutans* despite having an intermediate total phenolic content. This suggests that antimicrobial performance was influenced by the qualitative composition of the extracts and by possible combined effects among phenolic acids, flavonoids, anthocyanins, organic acids and other matrix components, rather than by total phenolics alone. Similar complexity has been reported for tomato pomace, in which polyphenol and carotenoid profiles were characterised, but antimicrobial activity was only modest overall [45].

Organic acids may also have contributed to the antibacterial response. In particular, ‘Heart’ M0% presented high levels of total organic acids, especially malic and tartaric acids, which could have favoured acid-related stress in bacterial cells. At the high extract concentrations required for some samples, nonspecific physicochemical effects, such as acidity, osmotic pressure, or total solids content of the crude extract, may also have contributed to growth inhibition. Therefore, the antimicrobial effect cannot be exclusively attributed to specific bioactive compounds [46].

*E. coli* also showed sensitivity to all extracts, with MICs ranging from 5.21 to 31.46 mg/mL. The lowest values were observed for ‘Round black’ M100% and ‘Round green’ M0%, suggesting that these accessions contained metabolites with a greater inhibitory effect against this bacterium, which may be partly related to their higher anthocyanin content at full ripeness, together with chlorogenic acid, *m*-coumaric acid, kaempferol and quercetin derivatives [47]. In contrast, *S. aureus* and *P. aeruginosa* exhibited higher MICs across several samples, with values near 83 mg/mL. This behaviour indicates weaker antibacterial activity against these strains, particularly against *P. aeruginosa*, whose outer membrane and resistance systems limit the action of many natural antimicrobials [43,48].

Recent work on tomato pomace reported that carotenoid extracts exhibited greater activity against *S. aureus* than against *E. coli*, whereas β-carotene required relatively high concentrations to inhibit bacteria. In the present study, the accessions and ripening stages with higher carotenoid accumulation should therefore be discussed as potentially contributing to antimicrobial activity, especially against Gram-positive bacteria, but not as the sole explanatory factor [45].

The absence of activity against *C. albicans* and *C. tropicalis* indicates that the extracts exhibited no detectable antifungal effect within the evaluated concentration range. This difference between bacteria and fungi can be explained by the structural characteristics of yeasts, whose cell wall and sterol-rich membrane may limit the action of crude plant extracts [9]. For these reasons, additional assays using higher concentrations, different solvents, and fractionated extracts can be adopted to further evaluate their potential activity. Finally, it should be noted that research primarily concentrates on in vitro antibacterial activity; conversely, evidence regarding in vivo antifungal and antiviral efficacy is scarce.

### 4.6. Statistical Analysis

The samples ‘Heart’ at M50% and M100% and ‘Red oval’ at M100% are clustered in the upper region of the dendrogram and simultaneously exhibit high levels of lycopene, β-carotene and various phenolic compounds, whilst showing lower levels of chlorophylls. This pattern is consistent with the physiological process of tomato ripening, characterised by the progressive degradation of chloroplasts and the formation of carotenoid-rich chromoplasts. Thus, other authors have demonstrated that the accumulation of lycopene and β-carotene occurs in a coordinated manner during ripening and is regulated by key genes in the carotenoid pathway, which explains the simultaneous appearance of these pigments in ripe fruits [36]. This has been confirmed by gene expression analyses in tomato with high lycopene content, in which increased expression of upstream pathway genes (GGPPS, PSY2, ZDS, and CrtISO), together with low expression of downstream genes (beta-LCY2 and epsilon-LCY), favours metabolic flux towards the simultaneous accumulation of both pigments. This process is hormonally orchestrated by ethylene signaling, which activates ripening-specific transcription factors [49,50].

In contrast, the ‘Round green’ samples are clearly distinct from the other accessions and are characterised by relatively higher levels of chlorophyll a and chlorophyll b. This indicates that their compositional profile is markedly different from that of the other accessions and changes very little throughout ripening; in other words, this accessions does not follow the same biochemical trajectory as the rest. At the structural level, this chlorophyll retention is associated with an incomplete or delayed chloroplast-to-chromoplast transition, a process that normally involves the disorganisation of thylakoid membranes and the reduction in the abundance of photosynthesis-associated proteins. When this process is limited, chloroplasts remain morphologically and functionally active even at advanced stages of fruit development, which may explain the distinct and stable position of green in the principal-component space of Figure 3, regardless of maturity stage [49,50].

Furthermore, a clustering of phenolic compounds—such as caffeic acid, chlorogenic acid, quercetin, quercetin glucoside and kaempferol—is observed alongside the ABTS and DPPH variables. This proximity suggests joint behaviour, as noted by other authors who have demonstrated that flavonoids and phenolic acids are important contributors to antioxidant capacity [32,42]. This association has been quantified in studies directly evaluating the radical-scavenging activity of individual phenolic acids and flavonoids, in which compounds such as caffeic acid, gallic acid and quercetin derivatives show IC50 values comparable to, or even lower than, those of reference antioxidants in DPPH and ABTS assays. Consistently, multivariate analyses of fruits have reported significant correlations between total polyphenol/flavonoid content and antioxidant capacity measured by both assays, supporting the interpretation that the clustering observed in the PCA reflects a functional relationship rather than merely a statistical association. However, because ABTS and DPPH are chemical radical-scavenging assays, this PCA association does not necessarily imply that the same compounds are responsible for the observed antimicrobial activity, nor that they exert equivalent biological activity in vivo [46,47].

Another clustering is observed among the organic acids (citric, malic, and tartaric), which show similar patterns across variables and are projected in similar directions in several panels, opposite to soluble solids. This behaviour is consistent with their involvement in respiratory metabolism and the tricarboxylic acid cycle, both of which undergo significant changes during fruit development and ripening, and it defines an axis of variation linked to the acid/sugar balance. In tomatoes, citric acid is quantitatively the predominant organic acid during most stages of fruit development, whereas malic acid becomes proportionally more relevant in green and immature fruits. Both acids participate in interconversion reactions within the tricarboxylic acid cycle, which explains why they tend to covary and separate from other variables in the multivariate analysis. The coordinated variation in these compounds directly influences acidity, flavour and sensory quality in tomato [42], although the exact contribution of each acid may vary among varieties and maturity stages [49,50].

Furthermore, principal component analysis showed similar vector orientations and multivariate associations among several variables between vitamin C and the weight and size of the fruit; between pH and quercetin, malic acid and sodium; between titratable acidity and chlorophyll B and naringenin; α-carotene with lycopene, *m*-coumaric acid and vitamin C; β-carotene with lycopene, *m*-coumaric acid, DPPH and vitamin C; lycopene with *m*-coumaric acid, DPPH and vitamin C; zeinoxanthin with *p*-coumaric acid, caffeic acid and naringenin; pheophytin with calcium; 4-hydroxybenzoic acid with DPPH and vitamin C; *p*-coumaric acid with caffeic acid and naringenin; *m*-coumaric acid with DPPH and vitamin C; chlorogenic acid with anthocyanins; ferulic acid with malic acid and tartaric acid; quercetin glycoside with quercetin and malic acid; quercetin with malic acid and sodium; ABTS with DPPH, potassium, magnesium and vitamin C; DPPH with potassium, magnesium and vitamin C; malic acid with tartaric acid and sodium; tartaric acid with sodium; potassium with magnesium. Thus, studies have demonstrated a direct relationship between the pigmented substances in tomatoes and their free radical-scavenging capacity (FRAP, DPPH, and ABTS) [32]. A positive association between β-carotene and lycopene has also been reported in previous studies [51,52]. Furthermore, a study of ten cherry tomato cultivars—six medium-sized and two small—at the red-ripe stage indicated a positive correlation between vitamin C content, total carotenoids, and DPPH [53]. Furthermore, other authors have demonstrated a positive correlation between total phenolic compounds and ascorbic acid content [51].

An inverse relationship was found between pH and titratable acidity, naringenin, potassium and magnesium; between ash and iron; between pheophytin and gallic acid and syringic acid; between chlorophyll b and sodium; between syringic acid and anthocyanins; between *m*-coumaric acid and tartaric acid; naringin with quercetin and sodium; ferulic acid with ABTS, DPPH, potassium, magnesium and vitamin C; quercetin glucoside with potassium and magnesium; quercetin with potassium and magnesium; ABTS with malic and tartaric acids; DPPH with malic and tartaric acids; malic acid with potassium and magnesium; tartaric acid with potassium, magnesium and vitamin C; potassium with sodium; magnesium with sodium. On the other hand, during tomato ripening, chlorophyll degradation occurs simultaneously with the accumulation of lycopene and β-carotene. The antagonism between chlorophylls and carotenoids has been extensively documented during the transition from chloroplasts to chromoplasts in climacteric fruits [36].

An additional pattern is the relatively independent arrangement of mineral variables (K, Ca, Mg, Fe, and Na) with respect to the main axis defined by pigments and phenolic compounds in several panels of Figure 4. This suggests that mineral composition responds, at least in part, to factors other than ripening, possibly including soil conditions or agronomic management. Nevertheless, potassium and calcium have been consistently associated with fruit-quality attributes, such as firmness and soluble solids content, through their roles in osmotic regulation and cell wall structure. This may explain why these variables tend to correlate closely with physical parameters, such as fruit weight and longitudinal and equatorial diameters, in some panels [43,47].

The biplots also show that soluble solids, titratable acidity, fruit weight and some mineral variables are not always aligned with the antioxidant variables, indicating that nutritional, physicochemical and antioxidant traits do not necessarily change in the same direction. This behaviour is expected in tomato fruit because ripening involves simultaneous but partially independent changes in sugars, acids, pigments, minerals, and secondary metabolites. Therefore, PCA is useful for visualising integrated quality patterns, but it should not be interpreted as a single maturity gradient for all compounds [50].

The PCA results also help to contextualise the antimicrobial response, although they should not be used to attribute this activity to a single compound. The lowest minimum inhibitory concentration (MIC) values reported in Table 7 corresponded to ‘Heart’ M0%, ‘Red oval’ M0% and ‘Round black’ M100%. These samples occupy distinct multivariate regions and exhibit distinct chemical profiles, suggesting that antimicrobial activity was likely influenced by a combination of compounds rather than by total phenolic content alone. This is consistent with the behaviour of crude plant extracts, in which phenolics, flavonoids, alkaloids, terpenes, peptides, organic acids and other metabolites may act through multiple mechanisms, including membrane effects, quorum-sensing interference or efflux-pump modulation [54].

At the molecular level, the antagonism between chlorophylls and carotenoids observed in the PCA—that is, chlorophyll degradation occurring simultaneously with the accumulation of lycopene and β-carotene—has been extensively documented during the chloroplast-to-chromoplast transition in climacteric fruits [36]. This coordination is regulated, at least in part, by master ripening transcription factors, such as RIN and NOR, and by ethylene signaling, which simultaneously activate genes of the carotenoid pathway and repress chlorophyll retention. Likewise, the clear separation of the five accessions observed in Figure 3, which is maintained even when maturity stages are contrasted, is consistent with studies that use principal component analysis as an effective tool for differentiating local tomato varieties based on their compositional and functional profiles [55,56].

In the particular case of ‘Round black’ M100%, the separation observed in the PCA may be partly related to variables associated with anthocyanins and flavonoids. This interpretation is supported by studies on tomato by-products, which show that dark-pigmented tomato materials may be enriched in flavonoids and anthocyanins and exhibit higher antioxidant capacity. However, this relationship should be presented as a compositional association and not as evidence that anthocyanins were the antimicrobial agents, because no bioassay-guided fractionation was performed [55,56].

Overall, the results indicate that accession identity and ripening stage jointly shape the physicochemical and compositional characteristics of cherry tomato fruit. The common cultivation environment allowed the accessions to be compared under similar general conditions, but the absence of genetic authentication, multilocation trials, and repeated cultivation cycles prevents the observed variation from being partitioned into genetic, environmental, and genotype-by-environment components. Therefore, the novelty of the study does not derive solely from the Ecuadorian origin of the samples. Rather, it lies in showing that differently coloured and shaped cherry tomato accessions cultivated under the same field context followed distinct compositional trajectories during ripening, with implications for accession-specific harvest selection and future germplasm characterisation.

## 5. Conclusions

The tomato is one of the most widely consumed vegetables worldwide; however, cherry tomatoes have gained increasing prominence in international markets due to their sensory, nutritional and functional characteristics. In this study, the results showed that the ‘Heart’ cherry tomatoes had the highest fruit weight and size, whilst potassium was the predominant mineral across all accessions assessed. Furthermore, vitamin C, carotenoids, anthocyanins, and phenolic compounds exhibited accumulation patterns that depended on both accessions and ripeness stage. Ripening increased vitamin C, carotenoids, and antioxidant activity in several accessions, notably ‘Heart’, ‘Red oval’, and ‘Round black’. In particular, ‘Heart’ and ‘Red oval’ reached the highest concentrations of vitamin C at the M100% ripeness stage, whilst ‘Round black’ exhibited the highest accumulation of anthocyanins, as well as high concentrations of carotenoids and flavonoids. Similarly, the ‘Round black’, ‘Red oval’ and ‘Heart’ cherry tomatoes stood out for their accumulation of lycopene and β-carotene in advanced stages of ripening. In contrast, ‘Round green’ maintained a profile characterised by high chlorophyll concentrations and low carotenoid content, reflecting a distinct metabolic strategy associated with its green colour. Antioxidant activity was associated with the combined accumulation of vitamin C, carotenoids, and phenolic compounds, reaching maximum values of 4.7 mmol TE/100 g DW by the DPPH assay and 3.6 mmol TE/100 g DW by the ABTS assay. Furthermore, the ethanolic extracts exhibited antimicrobial activity against *Escherichia coli*, *Staphylococcus aureus*, *Pseudomonas aeruginosa* and *Streptococcus mutans*, with minimum inhibitory concentrations ranging from 1.95 to 83.96 mg/mL. However, they showed no activity against *Candida albicans* or *Candida tropicalis*. Thus, the results indicate that the selection of the accessions and ripeness stage is an important factor in determining the nutritional and functional characteristics of cherry tomatoes.

## Figures and Tables

**Table 1:** Market quality of five cherry tomato accessions at three ripening stages.

Cherry Tomatoes	Maturity	Weight (g)	LD (mm)	ED (mm)	pH	SS (°Brix)	TA (%)	Humidity (%)	Ash (%)
‘Yellow oval’	M0%	9.0 ± 1.5 ^a^	21.1 ± 0.9 ^b^	33.2 ± 3.5 ^a^	4.3 ± 0.1 ^a^	5.1 ± 0.0 ^b^	0.8 ± 0.2 ^a^	85.7 ± 3.5 ^b^	1.1 ± 0.6 ^a^
	M50%	9.0 ± 0.3 ^a^	21.9 ± 0.8 ^b^	32.5 ± 0.8 ^a^	4.2 ± 0.0 ^b^	5.7 ± 0.5 ^b^	0.4 ± 0.1 ^c^	86.3 ± 0.4 ^b^	1.2 ± 0.3 ^a^
	M100%	11.8 ± 2.0 ^a^	35.8 ± 2.0 ^a^	21.4 ± 0.5 ^b^	3.8 ± 0.0 ^c^	7.3 ± 0.5 ^a^	0.7 ± 0.1 ^b^	93.8 ± 0.4 ^a^	0.8 ± 0.0 ^a^
‘Round green’	M0%	16.0 ± 2.8 ^a^	27.7 ± 0.7 ^a^	32.8 ± 1.4 ^a^	3.5 ± 0.0 ^a^	6.7 ± 0.5 ^a^	0.8 ± 0.1 ^a^	93.3 ± 0.9 ^a^	0.9 ± 0.0 ^a^
	M50%	12.9 ± 3.8 ^a^	25.4 ± 3.0 ^a^	30.8 ± 3.0 ^a^	3.5 ± 0.0 ^a^	4.7 ± 0.2 ^c^	0.9 ± 0.0 ^a^	92.3 ± 0.0 ^b^	1.0 ± 0.0 ^a^
	M100%	13.8 ± 3.0 ^a^	25.7 ± 1.2 ^a^	31.0 ± 4.1 ^a^	3.3 ± 0.0 ^b^	5.7 ± 0.5 ^b^	0.9 ± 0.1 ^a^	93.3 ± 0.2 ^a^	0.9 ± 0.0 ^a^
‘Round black’	M0%	13.1 ± 0.6 ^a^	29.6 ± 1.3 ^a^	28.3 ± 0.5 ^a^	3.7 ± 0.0 ^b^	5.0 ± 0.5 ^ab^	0.6 ± 0.0 ^a^	92.1 ± 0.2 ^a^	0.7 ± 0.0 ^b^
	M50%	10.9 ± 3.6 ^a^	27.5 ± 2.3 ^a^	26.9 ± 3.1 ^a^	3.9 ± 0.0 ^a^	4.7 ± 0.2 ^b^	0.5 ± 0.0 ^b^	92.0 ± 2.4 ^a^	0.2 ± 0.0 ^c^
	M100%	13.5 ± 1.4 ^a^	28.9 ± 0.7 ^a^	29.9 ± 1.4 ^a^	3.6 ± 0.0 ^c^	5.4 ± 0.4 ^a^	0.5 ± 0.0 ^b^	91.2 ± 3.6 ^a^	0.9 ± 0.0 ^a^
‘Red oval’	M0%	6.1 ± 0.9 ^c^	19.9 ± 2.0 ^b^	28.5 ± 2.7 ^b^	4.4 ± 0.4 ^a^	6.3 ± 0.5 ^a^	0.3 ± 0.1 ^b^	93.5 ± 3.1 ^a^	1.2 ± 0.1 ^a^
	M50%	10.2 ± 1.2 ^b^	23.5 ± 1.8 ^b^	33.3 ± 0.5 ^a^	4.1 ± 0.0 ^a^	6.4 ± 0.3 ^a^	0.4 ± 0.0 ^ab^	91.7 ± 1.6 ^a^	0.7 ± 0.4 ^a^
	M100%	14.6 ± 2.0 ^a^	28.5 ± 1.8 ^a^	33.3 ± 1.5 ^a^	4.2 ± 0.0 ^a^	6.3 ± 0.2 ^a^	0.4 ± 0.1 ^a^	88.7 ± 2.7 ^a^	1.2 ± 0.2 ^a^
‘Heart’	M0%	41.2 ± 3.5 ^b^	50.1 ± 3.1 ^a^	36.9 ± 1.8 ^b^	4.3 ± 0.0 ^a^	5.2 ± 0.2 ^b^	0.4 ± 0.0 ^a^	91.7 ± 3.6 ^a^	2.5 ± 0.0 ^a^
	M50%	67.3 ± 11.2 ^a^	46.3 ± 4.5 ^a^	54.9 ± 7.5 ^a^	4.1 ± 0.0 ^b^	6.3 ± 0.2 ^a^	0.3 ± 0.0 ^a^	83.0 ± 13.3 ^a^	0.8 ± 0.2 ^b^
	M100%	71.1 ± 9.5 ^a^	46.3 ± 5.2 ^a^	64.8 ± 4.8 ^a^	4.2 ± 0.0 ^b^	6.3 ± 0.5 ^a^	0.2 ± 0.2 ^a^	84.9 ± 4.9 ^a^	2.2 ± 0.5 ^a^
A_M0%_		***	***	***	***	***	***	**	***
A_M50%_		***	***	***	***	***	***	*	***
A_M100%_		***	***	***	***	***	***	**	***

Note: LD, longitudinal diameter; ED, equatorial diameter; SS: soluble solids; TA: total titratable acidity. Data are expressed as the mean ± standard deviation. Lowercase letters next to standard deviation values indicate significant differences between maturity stages within the same accessions. A_M0%_, Statistical comparison between all accessions at 0% maturity; A_M50%_, Statistical comparison between all accessions at 50% maturity; and A_M100%_, Statistical comparison between all accessions at 100% maturity. Significance levels are indicated as: ***, *p* < 0.0005; **, *p* < 0.005; *, *p* < 0.05.

**Table 2:** Mineral profile of five cherry tomato accessions at three ripening stages.

Cherry Tomatoes	Maturity	Ca (mg/100 DW)	Fe (mg/100 g DW)	K (mg/100 g DW)	Mg (mg/100 g DW)	Na (mg/100 g DW)
‘Yellow oval’	M0%	733.4 ± 64.0 ^a^	nd	2345.8 ± 6.2 ^b^	603.0 ± 1.6 ^b^	26.0 ± 5.7 ^c^
	M50%	448.5 ± 97.6 ^b^	5.9 ± 1.03 ^a^	1973.3 ± 123.1 ^c^	601.2 ± 0.2 ^b^	64.5 ± 0.8 ^a^
	M100%	300.4 ± 14.2 ^c^	4.5 ± 0.4 ^b^	3899.7 ± 17.2 ^a^	1059.7 ± 22.3 ^a^	41.3 ± 1.2 ^b^
‘Round green’	M0%	249.9 ± 11.1 ^b^	4.3 ± 0.2 ^a^	3608.6 ± 100.7 ^c^	1402.1 ± 73.9 ^a^	6.3 ± 0.8 ^b^
	M50%	267.9 ± 1.2 ^a^	5.1 ± 0.5 ^a^	5263.7 ± 59.8 ^a^	1432.4 ± 26.2 ^a^	4.6 ± 0.5 ^b^
	M100%	219.8 ± 4.9 ^c^	2.8 ± 0.5 ^b^	4016.7 ± 161.6 ^b^	1509.6 ± 82.2 ^a^	9.1 ± 1.2 ^a^
‘Round black’	M0%	325.4 ± 0.5 ^b^	3.2 ± 0.0 ^b^	3820.0 ± 121.4 ^b^	1447.8 ± 112.1 ^b^	27.4 ± 0.0 ^b^
	M50%	350.8 ± 3.5 ^a^	9.2 ± 0.6 ^a^	4058.3 ± 58.5 ^a^	1619.8 ± 79.9 ^a^	42.6 ± 2.5 ^a^
	M100%	289.3 ± 4.5 ^c^	3.1 ± 0.1 ^b^	3760.4 ± 15.1 ^b^	1246.5 ± 8.5 ^c^	14.6 ± 0.7 ^c^
‘Red oval’	M0%	245.7 ± 29.9 ^b^	nd	2124.1 ± 28.4 ^b^	563.4 ± 11.6 ^b^	74.4 ± 4.4 ^a^
	M50%	227.7 ± 4.3 ^b^	nd	1833.6 ± 103.3 ^b^	491.2 ± 11.6 ^b^	74.8 ± 3.3 ^a^
	M100%	329.2 ± 27.2 ^a^	3.3 ± 0.0 ^a^	4806.6 ± 648.8 ^a^	1617.1 ± 93.1 ^a^	36.0 ± 5.5 ^b^
‘Heart’	M0%	269.3 ± 17.0 ^b^	nd	2317.9 ± 82.3 ^b^	636.7 ± 125.7 ^c^	81.4 ± 3.8 ^a^
	M50%	273.6 ± 4.9 ^b^	6.3 ± 0.3 ^a^	3669.9 ± 100.1 ^a^	1251.2 ± 12.1 ^a^	73.2 ± 4.6 ^a^
	M100%	318.4 ± 26.8 ^a^	nd	3301.3 ± 487.9 ^a^	886.2 ± 114.3 ^b^	42.1 ± 4.8 ^b^
A_M0%_		***	***	***	***	***
A_M50%_		***	***	***	***	***
A_M100%_		***	***	***	***	***

Note: nd, undetectable; data are expressed as the mean ± standard deviation. Lowercase letters next to standard deviation values indicate significant differences between maturity stages within the same accessions. A_M0%_, Statistical comparison between all accessions at 0% maturity; A_M50%_, Statistical comparison between all accessions at 50% maturity; and A_M100%_, Statistical comparison between all accessions at 100% maturity. Significance levels are indicated as: ***, *p* < 0.0005 Calcium showed the highest concentration in the ‘Yellow oval’ cherry tomatoes, with its content decreasing as ripening progressed. A similar trend was observed in the ‘Heart’ cherry tomatoes. Potassium was the most abundant mineral across all tomatoes studied and, in most cases, reached its highest concentration at the M50% ripeness stage, except in the ‘Yellow oval’ and ‘Red oval’ cherry tomatoes.

**Table 3:** Vitamin C, organic acid and total anthocyanin content in five cherry tomato accessions at three ripening stages.

Cherry Tomatoes	Maturity	Vitamin C (mg/100 g DW)	Citric Acid (mg/100 g DW)	Malic Acid (mg/100 g DW)	Tartaric Acid (mg/100 g DW)	Total Organic Acid (mg/100 g DW)	Total Anthocyanin (mg/100 g DW)
‘Yellow oval’	M0%	107.2 ± 4.6 ^b^	1100.6 ± 80.2 ^c^	1795.8 ± 85.8 ^a^	497.1 ± 15.9 ^b^	3393.6 ± 181.9 ^b^	16.3 ± 0.9 ^a^
	M50%	14.0 ± 0.2 ^c^	3359.0 ± 305.6 ^a^	1742.0 ± 120.3 ^a^	765.5 ± 34.7 ^a^	5866.5 ± 391.2 ^a^	8.3 ± 1.8 ^b^
	M100%	236.5 ± 19.3 ^a^	2421.8 ± 83.4 ^b^	596.8 ± 26.9 ^b^	117.3 ± 2.1 ^c^	3135.9 ± 112.3 ^b^	9.0 ± 0.2 ^b^
‘Round green’	M0%	51.3 ± 2.6 ^a^	2807.8 ± 88.9 ^a^	406.6 ± 10.6 ^a^	376.5 ± 17.3 ^ab^	3590.9 ± 95.5 ^a^	13.0 ± 3.3 ^ab^
	M50%	43.6 ± 0.5 ^b^	2438.0 ± 414.6 ^a^	322.1 ± 58.7 ^a^	412.5 ± 24.1 ^a^	3172.5 ± 49.7 ^b^	17.2 ± 0.6 ^a^
	M100%	44.9 ± 2.3 ^b^	2316.6 ± 79.1 ^a^	378.5 ± 74.5 ^a^	359.8 ± 20.4 ^b^	3054.9 ± 15.8 ^c^	12.2 ± 2.3 ^b^
‘Round black’	M0%	101.3 ± 0.9 ^c^	1675.3 ± 71.5 ^b^	332.5 ± 2.5 ^c^	215.7 ± 4.2 ^a^	2223.5 ± 73.3 ^b^	12.7 ± 1.7 ^c^
	M50%	171.4 ± 11.2 ^b^	2073.4 ± 37.3 ^a^	451.5 ± 11.1 ^b^	212.6 ± 13.2 ^a^	2737.4 ± 61.6 ^a^	33.5 ± 2.6 ^b^
	M100%	234.7 ± 24.5 ^a^	2008.6 ± 42.1 ^a^	566.4 ± 35.2 ^a^	215.9 ± 1.9 ^a^	2790.9 ± 8.9 ^a^	46.2 ± 4.1 ^a^
‘Red oval’	M0%	11.8 ± 1.0 ^c^	1015.2 ± 30.7 ^c^	1958.2 ± 56.3 ^a^	729.4 ± 58.4 ^a^	3702.8 ± 145.4 ^b^	12.6 ± 1.7 ^a^
	M50%	39.0 ± 5.8 ^b^	1594.9 ± 71.3 ^b^	1801.8 ± 12.6 ^b^	783.2 ± 40.4 ^a^	4180.0 ± 18.3 ^a^	15.8 ± 0.7 ^a^
	M100%	428.7 ± 4.1 ^a^	2788.3 ± 154.2 ^a^	407.4 ± 24.3 ^c^	37.7 ± 2.2 ^b^	3233.4 ± 180.6 ^c^	13.0 ± 3.6 ^a^
‘Heart’	M0%	117.8 ± 8.5 ^c^	1273.4 ± 12.3 ^b^	1727.6 ± 55.3 ^a^	979.7 ± 2.0 ^a^	3980.6 ± 41.1 ^a^	10.8 ± 1.2 ^a^
	M50%	320.2 ± 1.6 ^b^	1355.1 ± 92.3 ^b^	324.7 ± 22.2 ^b^	261.3 ± 25.8 ^b^	1941.1 ± 140.2 ^c^	6.4 ± 1.2 ^b^
	M100%	469.3 ± 7.2 ^a^	1864.7 ± 3.3 ^a^	326.7 ± 3.7 ^b^	156.7 ± 14.7 ^c^	2348.2 ± 7.8 ^b^	7.5 ± 1.2 ^b^
A_M0%_		***	***	***	***	***	**
A_M50%_		***	***	***	***	***	***
A_M100%_		***	***	***	***	***	***

Note: Data are expressed as the mean ± standard deviation. Lowercase letters next to standard deviation values indicate significant differences between maturity stages within the same accessions. A_M0%_, Statistical comparison between all accessions at 0% maturity; A_M50%_, Statistical comparison between all accessions at 50% maturity; and A_M100%_, Statistical comparison between all accessions at 100% maturity. Significance levels are indicated as: ***, *p* < 0.0005; **, *p* < 0.005.

**Table 4:** Carotenoid profile and chlorophyll and derivatives of five cherry tomato accessions at three ripening stages.

Cherry Tomatoes	Maturity	Carotenoid Profile (mg/100 g DW)							Total Chlorophyll and Derivatives (mg/100 g DW)		
		α-Carotene	β-Carotene	Lutein	Zeaxanthin	β-Criptoxanthin	Lycopene	Total Carotenoids	Pheophytin b	Chlorophyll b	Total Chlorophyll and Derivatives
‘Yellow oval’	M0%			6.2 ± 1.0 ^a^	0.3 ± 0.0 ^a^			6.4 ± 1.0 ^b^	74.4 ± 1.1 ^a^	0.3 ± 0.0 ^a^	74.8 ± 1.1 ^a^
	M50%			0.1 ± 0.0 ^c^				0.1 ± 0.0 ^c^	4.0 ± 2.0 ^b^		4.0 ± 2.0 ^b^
	M100%		8.5 ± 0.3 ^a^	1.5 ± 0.2 ^b^	0.2 ± 0.0 ^b^			10.3 ± 0.6 ^a^			
‘Round green’	M0%	3.0 ± 0.1 ^a^		0.2 ± 0.0 ^c^	0.7 ± 0.0 ^a^	1.2 ± 0.0 ^a^		5.1 ± 0.2 ^a^	61.2 ± 2.0 ^a^		61.2 ± 2.0 ^a^
	M50%			1.1 ± 0.0 ^a^	0.1 ± 0.0 ^b^			1.1 ± 0.0 ^b^	12.3 ± 0.5 ^b^	1.5 ± 0.1 ^a^	13.8 ± 0.5 ^b^
	M100%			0.7 ± 0.1 ^b^	0.1 ± 0.0 ^b^			0.7 ± 0.1 ^c^	9.0 ± 0.2 ^c^	1.1 ± 0.0 ^b^	10.1 ± 0.2 ^c^
‘Round black’	M0%			0.7 ± 0.0 ^c^				0.7 ± 0.0 ^c^	16.2 ± 0.8 ^b^		16.2 ± 0.8 ^b^
	M50%		6.8 ± 0.7 ^b^	8.5 ± 1.4 ^a^			38.7 ± 4.5 ^b^	54.0 ± 6.6 ^b^	45.4 ± 4.9 ^a^		45.4 ± 4.9 ^a^
	M100%		77.2 ± 0.3 ^a^	5.4 ± 0.3 ^b^			991.0 ± 7.2 ^a^	1073.7 ± 7.2 ^a^			
‘Red oval’	M0%			0.0 ± 0.0 ^c^				0.0 ± 0.0 ^c^	7.4 ± 0.3 ^a^		7.4 ± 0.3 ^a^
	M50%		6.4 ± 0.3 ^b^	0.4 ± 0.0 ^b^	0.1 ± 0.0 ^a^	1.5 ± 0.1 ^a^	15.2 ± 0.6 ^b^	23.6 ± 0.9 ^b^	1.4 ± 0.1 ^b^		1.4 ± 0.1 ^b^
	M100%	7.1 ± 0.2 ^a^	28.1 ± 0.0 ^a^	2.5 ± 0.0 ^a^			911.2 ± 1.2 ^a^	948.9 ± 1.0 ^a^			
‘Heart’	M0%			0.0 ± 0.0 ^c^				0.0 ± 0.0 ^c^	1.9 ± 0.0 ^a^		1.9 ± 0.0 ^a^
	M50%		43.2 ± 0.5 ^a^	6.3 ± 0.1 ^a^			362.1 ± 0.8 ^b^	411.5 ± 1.4 ^b^			
	M100%	5.6 ± 0.1 ^a^	39.0 ± 2.4 ^b^	2.7 ± 0.1 ^b^			1026.0 ± 2.8 ^a^	1073.3 ± 0.5 ^a^			
A_M0%_				***	***			***	***		***
A_M50%_			***	***	ns		***	***	***		***
A_M100%_		***	***	***	***		***	***			

Note: Data are expressed as the mean ± standard deviation. Lowercase letters next to standard deviation values indicate significant differences between maturity stages within the same accessions. A_M0%_, Statistical comparison between all accessions at 0% maturity; A_M50%_, Statistical comparison between all accessions at 50% maturity; and A_M100%_, Statistical comparison between all accessions at 100% maturity. Significance levels are indicated as: ***, *p* < 0.0005; ns, not significant.

**Table 5:** Phenolic profile (mg/100 g DW) of five cherry tomato accessions at three ripening stages.

Cherry Tomatoes	Maturity	Gallic Acid	4-Hydroxybenzoic Acid	*m*-Coumaric Acid	Syringic Acid	Chlorogenic Acid	Naringin	Caffeic Acid	Ferulic Acid	Kaempferol	Quercetin Glucoside	Quercetin	Total Phenolics
‘Yellow oval’	M0%	3.0 ± 1.0 ^b^	122.3 ± 4.3 ^b^		11.2 ± 1.5 ^c^	8.2 ± 1.0 ^b^			711.7 ± 72.1 ^a^	108.8 ± 10.6 ^a^	38.3 ± 3.9 ^b^	8.8 ± 1.7 ^a^	1012.4 ± 22.5 ^b^
	M50%	25.6 ± 2.3 ^a^	88.7 ± 2.1 ^b^		21.7 ± 0.3 ^b^				388.8 ± 5.0 ^b^	53.7 ± 4.0 ^a^	28.5 ± 0.4 ^c^	8.1 ± 4.4 ^a^	615.2 ± 1.2 ^c^
	M100%	23.0 ± 2.1 ^a^	861.7 ± 81.5 ^a^		36.9 ± 0.6 ^a^	22.9 ± 0.2 ^a^		287.2 ± 10.5 ^a^	110.9 ± 17.6 ^c^	37.3 ± 0.9 ^a^	57.6 ± 1.9 ^a^	10.3 ± 1.4 ^a^	1447.7 ± 11.1 ^a^
‘Round green’	M0%	16.5 ± 0.6 ^a^	104.1 ± 19.8 ^a^		11.6 ± 0.4 ^b^	16.5 ± 0.6 ^b^	287.7 ± 2.3 ^a^	329.6 ± 12.0 ^a^	134.7 ± 7.8 ^c^				905.9 ± 41.3 ^a^
	M50%	10.1 ± 0.0 ^b^	32.6 ± 4.5 ^c^		23.9 ± 1.7 ^a^	47.0 ± 0.2 ^a^	126.6 ± 5.2 ^c^	151.5 ± 5.5 ^b^	193.0 ± 5.0 ^a^				584.8 ± 12.0 ^c^
	M100%	9.9 ± 0.1 ^b^	57.0 ± 0.7 ^b^		24.8 ± 0.3 ^a^	47.6 ± 0.5 ^a^	212.5 ± 2.4 ^b^	135.9 ± 1.6 ^c^	150.3 ± 1.7 ^b^				638.1 ± 7.3 ^b^
‘Round black’	M0%	15.3 ± 2.3 ^b^	134.1 ± 15.8 ^c^	116.8 ± 17.8 ^b^	14.2 ± 0.2 ^a^	218.6 ± 4.2 ^a^				12.4 ± 1.0 ^c^	15.0 ± 1.2 ^c^	4.3 ± 0.5 ^c^	530.6 ± 42.6 ^b^
	M50%	15.9 ± 0.6 ^b^	159.7 ± 1.1 ^b^	206.5 ± 1.4 ^a^	9.6 ± 2.4 ^b^	223.3 ± 0.3 ^a^				36.1 ± 0.8 ^b^	22.5 ± 2.8 ^b^	8.3 ± 0.9 ^b^	682.1 ± 2.3 ^a^
	M100%	18.1 ± 0.6 ^a^	251.5 ± 2.4 ^a^	186.9 ± 4.6 ^a^	8.3 ± 1.5 ^b^	147.1 ± 3.1 ^b^				42.3 ± 0.5 ^a^	29.2 ± 1.3 ^a^	10.5 ± 0.3 ^a^	693.9 ± 9.5 ^a^
‘Red oval’	M0%	18.9 ± 0.5 ^c^	40.2 ± 0.6 ^c^		22.0 ± 0.5 ^a^	90.8 ± 3.8 ^b^			846.4 ± 25.0 ^a^	2.7 ± 0.0 ^c^	29.5 ± 2.7 ^b^	11.6 ± 1.4 ^b^	1062.2 ± 27.9 ^b^
	M50%	24.9 ± 3.1 ^b^	94.0 ± 9.6 ^b^		11.8 ± 0.3 ^b^	110.7 ± 5.5 ^a^		65.3 ± 5.6 ^a^	406.9 ± 33.6 ^b^	68.8 ± 5.4 ^a^	60.1 ± 1.7 ^a^	16.3 ± 0.9 ^a^	858.8 ± 1.6 ^c^
	M100%	33.3 ± 0.6 ^a^	448.4 ± 21.0 ^a^	698.2 ± 33.5 ^a^	24.2 ± 4.5 ^a^	65.3 ± 3.4 ^c^				44.0 ± 1.4 ^b^	7.8 ± 0.8 ^c^	7.8 ± 0.6 ^c^	1328.9 ± 23.9 ^a^
‘Heart’	M0%	21.1 ± 0.8 ^c^	132.7 ± 5.1 ^c^		18.7 ± 0.7 ^c^	74.9 ± 5.4 ^a^		46.0 ± 3.5 ^a^	240.0 ± 31.3 ^a^	3.4 ± 0.8 ^c^	34.6 ± 27.6 ^a^	13.4 ± 1.0 ^a^	584.7 ± 65.9 ^c^
	M50%	32.7 ± 2.3 ^a^	352.3 ± 17.3 ^b^	375.7 ± 11.9 ^a^	28.5 ± 1.0 ^b^	38.5 ± 0.4 ^b^				6.4 ± 0.6 ^b^	12.4 ± 0.8 ^a^	4.2 ± 1.1 ^b^	850.6 ± 31.8 ^b^
	M100%	29.6 ± 1.0 ^b^	565.5 ± 33.2 ^a^	366.3 ± 43.8 ^a^	30.8 ± 0.1 ^a^	33.8 ± 3.9 ^b^				25.3 ± 0.8 ^a^	8.7 ± 0.7 ^a^	3.9 ± 0.2 ^b^	1063.8 ± 78.4 ^a^
A_M0%_		***	***		***	***		***	***	*	*	***	***
A_M50%_		***	***	***	***	***		***	***	***	***	***	***
A_M100%_		***	***	***	***	***		***	**	***	***	***	***

Note: Data are expressed as the mean ± standard deviation. Lowercase letters next to standard deviation values indicate significant differences between maturity stages within the same accessions. A_M0%_, Statistical comparison between all accessions at 0% maturity; A_M50%_, Statistical comparison between all accessions at 50% maturity; and A_M100%_, Statistical comparison between all accessions at 100% maturity. Significance levels are indicated as: ***, *p* < 0.0005; **, *p* < 0.005; *, *p* < 0.05.

**Table 6:** Antioxidant activity of five cherry tomato accessions at three ripening stages.

Cherry Tomatoes	Maturity	DPPH (mmol TE/100 g DW)	ABTS (mmol TE/100 g DW)
‘Yellow oval’	M0%	1.3 ± 0.1 ^b^	2.4 ± 0.3 ^b^
	M50%	1.2 ± 0.1 ^b^	1.8 ± 0.2 ^b^
	M100%	4.7 ± 0.2 ^a^	3.6 ± 0.4 ^a^
‘Round green’	M0%	1.8 ± 0.1 ^b^	2.8 ± 0.2 ^a^
	M50%	2.5 ± 0.2 ^a^	2.6 ± 0.3 ^a^
	M100%	1.9 ± 0.3 ^b^	2.7 ± 0.3 ^a^
‘Round black’	M0%	3.2 ± 0.2 ^b^	3.0 ± 0.1 ^a^
	M50%	4.0 ± 0.1 ^a^	3.4 ± 0.1 ^a^
	M100%	4.4 ± 0.4 ^a^	3.4 ± 0.7 ^a^
‘Red oval’	M0%	1.3 ± 0.2 ^c^	1.5 ± 0.3 ^b^
	M50%	1.6 ± 0.1 ^b^	2.0 ± 0.2 ^b^
	M100%	4.6 ± 0.1 ^a^	3.0 ± 0.2 ^a^
‘Heart’	M0%	1.6 ± 0.1 ^b^	1.7 ± 0.2 ^b^
	M50%	3.4 ± 0.2 ^a^	2.9 ± 0.3 ^a^
	M100%	3.5 ± 0.1 ^a^	3.2 ± 0.2 ^a^
A_M0%_		***	***
A_M50%_		***	***
A_M100%_		***	*

Note: Data are expressed as the mean ± standard deviation. Lowercase letters next to standard deviation values indicate significant differences between maturity stages within the same accessions. A_M0%_, Statistical comparison between all accessions at 0% maturity; A_M50%_, Statistical comparison between all accessions at 50% maturity; and A_M100%_, Statistical comparison between all accessions at 100% maturity. Significance levels are indicated as: ***, *p* < 0.0005; *, *p* < 0.05.

**Table 7:** Minimum inhibitory concentration of dry ethanolic extracts of five cherry tomato accessions at three ripening stages.

Cherry Tomatoes	Maturity	Minimum Inhibitory Concentration (mg/mL)					
		Bacterial Strains				Fungal Strains	
		*E. coli* ATCC 8739	*S. aureus* ATCC 6538P	*P. aeruginosa* ATCC 9027	*S. mutans* ATCC 25175	*C. albicans* ATCC 1031	*C. tropicalis* ATCC 13803
‘Yellow oval’	M0%	20.94	83.75	83.75	83.75	-	-
	M100%	20.99	83.96	83.96	-	-	-
‘Round green’	M0%	5.22	10.44	41.77	20.89	-	-
	M100%	13.35	13.35	26.71	13.35	-	-
‘Round black’	M0%	7.83	62.60	62.60	62.60	-	-
	M100%	5.21	83.44	83.44	5.21	-	-
‘Red oval’	M0%	10.46	83.65	83.65	2.61	-	-
	M100%	10.48	83.85	83.85	41.93	-	-
‘Heart’	M0%	7.81	-	62.50	1.95	-	-
	M100%	31.46	-	-	-	-	-

Note: -, non-active at the tested concentration.

## Data Availability

The original contributions presented in this study are included in the article. Further inquiries can be directed to the corresponding author.
